# A pH-Adjustable Tissue Clearing Solution That Preserves Lipid Ultrastructures: Suitable Tissue Clearing Method for DDS Evaluation

**DOI:** 10.3390/pharmaceutics12111070

**Published:** 2020-11-09

**Authors:** Shintaro Fumoto, Eriko Kinoshita, Keisuke Ohta, Kei-ichiro Nakamura, Tasuku Hirayama, Hideko Nagasawa, Die Hu, Kazuya Okami, Riku Kato, Shojiro Shimokawa, Naho Ohira, Koyo Nishimura, Hirotaka Miyamoto, Takashi Tanaka, Shigeru Kawakami, Koyo Nishida

**Affiliations:** 1 Graduate School of Biomedical Sciences, Nagasaki University, Nagasaki 852-8501, Japan; kinoshitaeriko@gmail.com (E.K.); bb55720002@ms.nagasaki-u.ac.jp (D.H.); bb55619005@ms.nagasaki-u.ac.jp (K.O.); bb55620003@ms.nagasaki-u.ac.jp (R.K.); shishojiro@gmail.com (S.S.); fm.n.eethgie.619@gmail.com (N.O.); koynishi0115@gmail.com (K.N.); hmiyamoto@nagasaki-u.ac.jp (H.M.); t-tanaka@nagasaki-u.ac.jp (T.T.); skawakam@nagasaki-u.ac.jp (S.K.); koyo-n@nagasaki-u.ac.jp (K.N.); 2 Division of Microscopic and Developmental Anatomy, Department of Anatomy, Kurume University School of Medicine, Kurume 830-0011, Japan; kohta@med.kurume-u.ac.jp (K.O.); ana2nkmr@med.kurume-u.ac.jp (K.-i.N.); 3 Laboratory of Pharmaceutical & Medicinal Chemistry, Gifu Pharmaceutical University, 1-25-4, Daigakunishi, Gifu 501-1196, Japan; hirayamat@gifu-pu.ac.jp (T.H.); hnagasawa@gifu-pu.ac.jp (H.N.)

**Keywords:** tissue optical clearing, biological imaging, three-dimensional imaging, adult animal, microscopy, gene delivery, liposome

## Abstract

Visualizing biological events and states to resolve biological questions is challenging. Tissue clearing permits three-dimensional multicolor imaging. Here, we describe a pH-adjustable tissue clearing solution, Seebest (SEE Biological Events and States in Tissues), which preserves lipid ultrastructures at an electron microscopy level. Adoption of polyethylenimine was required for a wide pH range adjustment of the tissue clearing solution. The combination of polyethylenimine and urea had a good tissue clearing ability for multiple tissues within several hours. Blood vessels stained with lipophilic carbocyanine dyes were deeply visible using the solution. Adjusting the pH of the solution was important to maximize the fluorescent intensity and suppress dye leakage during tissue clearing. The spatial distribution of doxorubicin and oxidative stress were observable using the solution. Moreover, spatial distribution of liposomes in the liver was visualized. Hence, the Seebest solution provides pH-adjustable, rapid, sufficient tissue clearing, while preserving lipid ultrastructures, which is suitable for drug delivery system evaluations.

## 1. Introduction

Imaging biological events and states is important to resolve biological questions. Many researchers have developed various in vitro and in vivo imaging techniques [1,2,3,4]. Multiscale imaging, from whole body to sub-organelle levels, is required for clarification. Results from cell cultures are often misrepresentative of in vivo phenomena [5]. However, live animal imaging has difficulties, especially in visualizing the submicron level. Ex vivo observation of tissues is suitable for three-dimensional imaging at a targeted moment in time course experiments and has various merits, such as reflecting in vivo phenomena, submicron imaging, and visualizing spatial relationships among biological events and states. Tissue optical clearing methods facilitate three-dimensional multicolor deep imaging using fluorescence, and have been widely applied to neurology and pharmaceutics [6,7,8,9,10,11,12,13,14,15,16,17,18,19,20,21].

Methodologies of tissue optical clearing can be categorized into electrophoretic methods and sequential immersion in tissue clearing solutions. A representative of the former is the CLARITY protocol, which has an extreme tissue clearing efficiency [6]. In CLARITY, once a tissue is infused with formaldehyde and hydrogel monomers, it polymerizes into a hydrogel. Then, electrophoresis with a high sodium dodecyl sulfate concentration is performed to extract lipids, which are the main reason for opacity. Overall, this protocol requires almost 10 days. Therefore, CLARITY is a cumbersome and time-consuming task. However, immersion in tissue clearing solutions can be performed without specialized devices. Among them, aqueous solutions are useful to avoid inactivation of fluorescent proteins. In 2011, Hama et al. developed a urea-based tissue clearing formulation, Sca*l*eA2, which optically cleared the brain in 2 weeks [7]. However, Sca*l*eA2 had some problems, including an insufficient tissue clearing efficiency, slow rate of tissue clearing, fragility, and swelling. Susaki et al. developed CUBIC (clear, unobstructed brain imaging cocktails and computational analysis), which greatly improved tissue clearing efficiency using high concentrations of a detergent, urea, and amino alcohols [8]. However, CUBIC affects the ultrastructure of specimens, mainly because of the high concentration of the detergent [11]. Hama et al. developed Sca*l*eS with urea and sorbitol, a mild tissue-permeant sugar alcohol [11]. Sca*l*eS contains minimal detergent and preserves ultrastructures. However, Sca*l*eS requires sequential immersion into a series of solutions (Sca*l*eS0, S1, S2, S3, and S4) and is slightly cumbersome and time consuming (more than 3 days). Chen et al. developed UBasM (Urea-Based Amino-Sugar Mixture), which contains urea, sugar amine meglumine, and a low concentration of detergent [12]. Here, even a low concentration of detergent can theoretically affect lipid structures. Detergent-free methods have also been developed. The *Clear^T2^* protocol includes 50% formamide and 10–20% polyethylene glycol [13]. SeeDB (See Deep Brain) uses an extremely high concentration of fructose (gradually increased from 20 to 115 *w*/*v*%) [14]. Unfortunately, the tissue clearing efficiencies of *Clear^T2^* and SeeDB for adult tissues are insufficient for deep imaging with confocal microscopy [16,19]. However, the combination of fructose and urea, such as in FRUIT [20] and FUnGI (fructose, urea, and glycerol for imaging) [21], appears to be a useful strategy. Researchers must select a tissue optical clearing method by compromising between transparency and ultrastructure preservation.

Not only amides such as urea, but also amines are often used in tissue clearing. CUBIC uses two amines, *N,N,N′,N′*-tetrakis(2-hydroxypropyl)ethylenediamine and 2,2′,2′′-nitrilotriethanol, and UBasM uses meglumine. Although these amines have a buffering capacity, a tissue clearing method applicable to various pHs is not available, even though many fluorescent dyes and proteins are dependent on pH. In this study, we focused on polyethylenimine (PEI), which has a unique buffering capacity over an appreciable range of pH [22]. Here, using PEI, we developed a novel tissue optical clearing solution, Seebest (SEE Biological Events and States in Tissues), which is a pH-adjustable method. To observe biological events and states using fluorescent dyes, it is preferable to preserve lipid structures to maintain barrier functions against leakage of fluorescent dyes. Therefore, in this study, Seebest without detergents was developed. Seebest provided sufficient transparency while preserving lipid ultrastructures.

## 2. Materials and Methods

### 2.1. Materials

PEI with average molecular weights of 600, 1800, and 10,000 was obtained from Wako Pure Chemical Industries, Osaka, Japan (Product number: 163-17835, 169-17815, and 166-17825, respectively). Propylene oxide-denatured PEI (PP-061) was kindly provided by Nippon Shokubai Co., Ltd., Tokyo, Japan).

### 2.2. Plasmid

pZsGreen1-N1 expressing a variant of the Zoanthus sp. green fluorescent protein, ZsGreen1, was purchased from Clontech Laboratories (Mountain View, CA, USA). pZsGreen1-N1 was amplified in Escherichia coli strain DH5α, isolated, and purified using an EndoFree Plasmid Giga Kit (Qiagen GmbH, Hilden, Germany). Plasmid DNA dissolved in water was stored at −20 °C prior to experiments.

### 2.3. Mice

Male ddY mice (5-week-old, 24.0–29.5 g) were obtained from Japan SLC, Inc. (Shizuoka, Japan). Anesthesia was induced by intraperitoneal injection of a mixture of 0.75 mg/kg medetomidine, 5 mg/kg butorphanol, and 4 mg/kg midazolam. Animal experiments were performed in accordance with the guidelines of Nagasaki University and approved by the university committee (Protocol Approval No. 1603101289). This work complied with all relevant ethical regulations regarding animal research.

### 2.4. Preparation of Seebest Solutions

Once PEI was dissolved in distilled water, the pH of the solution was adjusted with 8 M hydrochloric acid (Nacalai Tesque Inc., Kyoto, Japan, 18321-05) on ice to prevent colorization. Then, urea (Wako Pure Chemical Industries, 210-01185) was dissolved in the pH-adjusted PEI solution at approximately 60 °C using a hot stirrer. Finally, pH of the solution was finely adjusted at 30 °C. Typical final concentrations of PEI and urea were 20 *w*/*v*% and 8 M, respectively. Seebest-PP (Propylene oxide-denatured PEI) was prepared in a similar fashion except for the use of propylene oxide-denatured PEI instead of PEI. 4′,6-diamidino-2-phenylindole (DAPI) (Sigma Aldrich Co. LLC., Saint Louis, MO, USA) was optionally added to the Seebest solution at a concentration of 5 µg/mL. It was necessary to use freshly prepared Seebest due to the stability and urea crystallization.

### 2.5. Tissue Clearing in Seebest

After pre-warming the Seebest solution, a tissue specimen was set in the solution and incubated at 37 °C. Incubation time was 1 day unless otherwise indicated. During incubation, a tube rotator was used to stir the solution.

### 2.6. Comparison with Other Clearing Methods

Each clearing method was performed in accordance with the literature (Sca*l*eA2 [7], Sca*l*eS, and Sca*l*eSQ(0) [11], CUBIC [8], *Clear^T2^* [12], and FUnGI [21]). The composition of each solution was as follows. Sca*l*eA2, urea (4 M), glycerol (10.0 *w*/*v*%), and Triton x-100 (0.1 *v*/*v*%). Sca*l*eS0, d-(−)-sorbitol (20 *w*/*v*%), glycerol (5 *w*/*v*%), methyl-β-cyclodextrin (1 mM), γ-cyclodextrin (1 mM), *N*-acetyl-L-hydroxyproline (1 *w*/*v*%), and dimethyl sulfoxide (DMSO, Nacalai Tesque, 13445-45) (3 *v*/*v*%) in PBS(−)). Sca*l*eS1, d-(−)-sorbitol (20 *w*/*v*%), glycerol (10 *w*/*v*%), urea (4 M), and Triton x-100 (0.2 *w*/*v*%). Sca*l*eS2, d-(−)-sorbitol (27 *w*/*v*%), urea (2.7 M), Triton x-100 (0.1 *w*/*v*%), and DMSO (8.3 *v*/*v* %). Sca*l*eS3, d-(−)-sorbitol (36.4 *w*/*v*%), urea (2.7 M), and DMSO (9.1 *v*/*v*%). Sca*l*eS4, d-(−)-sorbitol (40 *w*/*v*%), glycerol (10 *w*/*v*%), urea (4 M), Triton x-100 (0.2 *w*/*v*%), and DMSO (15 *v/v%*). Sca*l*eSQ(0), D-(−)-sorbitol (22.5 *w*/*v*%) and urea (9.1 M). CUBIC-1, urea (25 *w*/*v*%), *N*,*N*,*N′*,*N′*-tetrakis(2-hydroxypropyl)ethylenediamine (25 *w*/*v*%), and Triton x-100 (15 *w*/*v*%). CUBIC-2, sucrose (50 *w*/*v*%), urea (25 *w*/*v*%), 2,2′,2″-nitrilotriethanol (10 *w*/*v*%) and Triton x-100 (0.1 *v/v%*). *Clear^T2^*, formamide (50 *v/v%*) polyethylene glycol (average molecular weight 8000, 20 *w*/*v*%). To prepare FUnGI, 10 g fructose was dissolved in a mixture of 2.33 mL Tris-EDTA (100 mM Tris and 10 mM EDTA disodium salt, pH 8.0) and 11 mL glycerol for 1 day at room temperature. Then, 3.31 g urea was added to the mixture and dissolved for further 1 day, producing approximately 20 mL FUnGI.

### 2.7. Confocal Microscopy

Cleared specimens were imaged under an inverted confocal laser scanning microscope (LSM710, Carl Zeiss Microscopy GmbH, Cologne, Germany). Laser wavelengths were 405, 488, 543, and 633 nm. Lenses were a ×10 EC Plan-Neofluar (numerical aperture (NA): 0.30; working distance (WD): 5.2 mm), ×20 EC Plan-Neofluar ×20 (NA: 0.50; WD: 2.0 mm), ×25 LD LCI Plan-Apochromat (NA: 0.8; WD: 0.57 mm), and ×40 LD C-Apochromat (NA: 1.1; WD: 0.62 mm). Images were acquired by Zen Black software (version 2012, Carl Zeiss Microscopy).

### 2.8. Transmission Electron Microscopy

Mice underwent hepatic perfusion fixation under anesthesia. Perfusates were 10 mL PBS (−) containing 10 U/mL heparin, 30 mL PBS (−) and 40 mL fixative in this order. For transmission electron microscopy, the fixative was 4% paraformaldehyde (PFA) with 0.1% glutaraldehyde (GA) in PBS (−). Then, the liver specimens were immersed in the same fixative overnight at 4 °C. The specimens were washed twice in PBS (−) and immersed in Seebest (pH 7.3 or 11) or PBS (−) for 1 day. After that, the specimens were washed twice in PBS (−) and incubated in PBS (−) for 1 day. The specimens were sectioned at 0.5 mm thicknesses, post-fixed with 1% osmium tetroxide in 0.1 M phosphate buffer (pH 7.4) for 2 h, dehydrated in acetone, and embedded in Epon812 resin (TAAB, Berkshire, UK). Ultrathin sections (70 nm thick) were prepared with a diamond knife, stained with uranyl acetate and lead citrate, and observed under a transmission electron microscope (H7650, Hitachi High-Technologies Corporation, Tokyo, Japan).

### 2.9. Dox Visualization

Doxorubicin (Dox) hydrochloride (Tokyo Chemical Industry Co., Ltd., Japan, D4193) was dissolved in DMSO as a stock solution (10 mg/mL). To measure Dox release during tissue clearing, the stock solution of Dox was diluted in saline at a concentration of 1 mg/mL and then intravenously injected into mice (10 mg/kg). Mice were subjected to cardiac perfusion fixation under anesthesia. Perfusates were 10 mL PBS (−) containing 10 U/mL heparin, 50 mL PBS (−), and 160 mL 4% PFA in this order. Then, the liver was cut into small blocks. The blocks of liver were weighed, and 20 µL/mg Seebest solutions (pH 5, 6, and 7) were added to the blocks. One day after incubation at 37 °C on the tube rotator, fluorescence in the external fluid was measured at excitation and emission wavelengths of 488 and 570 nm, respectively. For three-dimensional observation, the stock solution of Dox was diluted in saline at a concentration of 2 mg/mL, and then intravenously injected into mice (20 mg/kg). Cardiac perfusion was performed. Perfusates (filtrated with a 0.45 µm cellulose acetate filter) were 10 mL PBS (−) containing 10 U/mL heparin, 50 mL PBS (−), 5 mL 5% glucose, 10 mL 0.12 mg/mL 1,1′-dioctadecyl-3,3,3′,3′-tetramethylindodicarbocyanine (DiD) (perchlorate salt, Thermo Fisher Scientific Inc., Bedford, MA, USA, D307) in 5% glucose, 5 mL 5% glucose and 160 mL 4% PFA in this order. Then the heart, liver, and kidney were subjected to post-fixation at 4 °C overnight. Tissues were washed twice in PBS (−) and then immersed in Seebest solution at 37 °C. Confocal microscopy was performed within 4 h of immersion with Seebest.

### 2.10. Liver Ischemia/Reperfusion Injury

Reactive oxygen species (ROS) probe CellROX Deep Red (125 µmole, Thermo Fisher Scientific Inc., Bedford, MA, USA, C10422) was injected intraperitoneally. One hour after injection, mice were anesthetized, and the portal vein of mice was clamped for 30 min and then reperfused for 1 h. Intravenous injection of 1,1′-dioctadecyl-3,3,3′,3′-tetramethylindocarbocyanine (DiI) (perchlorate salt, Sigma Aldrich Co. LLC., 42364) in 5% glucose (0.5 mg/mL, 4.8 mg/kg) and perfusion fixation were performed. A liver specimen was subjected to post-fixation at 4 °C overnight. Tissues were washed twice in PBS (−) and then immersed in Seebest solution at 37 °C. Confocal microscopy was performed at 1 day after immersion in Seebest.

### 2.11. Hydrodynamics-Based In Vivo Transfection to the Liver

Mice were injected with a plasmid DNA (pZsGreen1-N1) solution in saline (10 µg/2.2 mL/mouse) within 5 sec via the tail vein. One hour prior to plasmid DNA injection, CellROX Deep Red (125 µmole) was injected intraperitoneally. Twelve hours after plasmid DNA injection, mice were intravenously injected with a DiI solution in 5% glucose (0.5 mg/mL, 4.8 mg/kg). Thirty minutes after DiI injection, mice underwent hepatic perfusion fixation. A liver specimen was cleared in Seebest (pH 8.5) for 1 day and then confocal microscopy was performed.

### 2.12. Preparation and Characterization of Liposomes for Spatial Distribution

Traditional Bangham and limit size liposomes were prepared as follows. Traditional Bangham liposomes were prepared using thin lipid film hydration (Bangham’s method). Egg lecithin (Wako Pure Chemical Industries, Osaka City, Osaka Prefecture, Japan 124-05031), cholesterol (Nacalai Tesque Inc., Kyoto, Japan 08721-62), and 3,3′-dioctadecyloxacarbocyanine perchlorate (DiO) (Sigma Aldrich Co. LLC., D4292) were dissolved in methanol at a weight ratio of 75:25:1.42. Then, a thin lipid film was formed using a rotary evaporator, which was subsequently desiccated overnight. To obtain liposomes, the thin lipid film was rehydrated in a 5% glucose solution at a final lipid concentration of 2 mg/mL. Liposomes were extruded through polycarbonate membrane filters (200-nm pore sizes, 11 times) using a commercially available instrument (Mini-Extruder, Avanti Polar Lipids, Inc., Alabaster, AL, USA). Limit size liposomes were prepared based on microfluidics using a NanoAssemblr instrument (Precision Nanosystems, Vancouver, BC, Canada). Briefly, anhydrous ethanol was prepared by dehydration of ethanol (Kanto Chemical Co., Inc., Tokyo, Japan, 14033-70) using a molecular sieve (3A, powder, Nacalai Tesque, 04176-55). Egg lecithin (7.5 mg), cholesterol (2.5 mg), and DiO (0.142 mg) were dissolved in anhydrous ethanol and evaporated using the rotary evaporator. Then, the lipids were again dissolved in 1 mL anhydrous ethanol, and this solution served as the ethanol phase. Glucose (Nacalai Tesque, 16806-25) was dissolved in distilled water at a concentration of 5 *w*/*v*%, and this solution served as the water phase. Ethanol and water phases (volume ratio 1:5) were mixed together using the NanoAssemblr instrument at a flow rate of 12 mL/min and dialyzed overnight using a dialyzing tube (Spectra/Por Float-A-Lyzer G2, 1 mL size, 3.5–5 KDa, Spectrum Laboratories Inc., Piscataway, NJ, USA) against a 5% glucose solution to produce limit size liposomes. The particle size of liposomes was measured using a Zetasizer Nano ZS instrument (Malvern Instruments Ltd., Worcestershire, UK).

### 2.13. Lipophilic Carbocyanine Dye Staining

To stain blood vessels, DiI perfusion was performed as reported previously [23] with slight modifications. Originally, DiI was dissolved in ethanol. However, dissolution rate of DiI in ethanol appeared to be slow. Before perfusion, the DiI stock solution (8 mg/mL in DMSO) was diluted in 5% glucose solution to produce a 0.12 mg/mL solution. DiD perfusion was performed in the same fashion.

### 2.14. Fixation

Fixation was performed by cardiac or hepatic perfusion under anesthesia. For cardiac perfusion, a bent needle (26G, Nipro Corporation, Osaka, Japan) equipped with a tube and a peristatic pump was inserted to the left ventricular. Then, right atrial was cut with surgical scissors. Immediately, perfusion was started. For hepatic perfusion, the portal vein was catheterized with a Surflo^®^ I.V. Catheter, 22 G × 1 1/4″ (Terumo Co., Tokyo, Japan). Immediately, the liver was perfused with perfusates. The fixative was 4% PFA in PBS (−), except for electron microscopy (4% PFA with 0.1% GA). Tissue specimens (indicated in each figure) were subjected to post-fixation at 4 °C overnight. Before tissue clearing in Seebest, tissue specimens were washed twice in PBS (−).

### 2.15. FRET Analysis for Integrity of Lipid Membranes

Förster resonance energy transfer (FRET) liposomes were prepared using thin lipid film hydration (Bangham’s method). Egg lecithin, cholesterol, 1,2-dioleoyl-sn-glycero-3-phosphoethanolamine-*N*-(7-nitro-2-1,3-benzoxadiazol-4-yl) (ammonium salt, Avanti Polar Lipids, 810145), and 1,2-dioleoyl-sn-glycero-3-phosphoethanolamine-*N*-(lissamine rhodamine B sulfonyl) (ammonium salt, Avanti Polar Lipids, 810150) were dissolved in methanol at a weight ratio of 100:50:2.4:3.4. Then, a thin lipid film was formed using a rotary evaporator and subsequently desiccated overnight. To obtain FRET liposomes, the thin lipid film was rehydrated in a 5% glucose solution at a final lipid concentration of 0.4 mg/mL. Liposomes were extruded through polycarbonate membrane filters (100- and 50-nm pore sizes, 11 times for each pore size) using a commercially available instrument (Mini-Extruder, Avanti Polar Lipids, Inc.). FRET relaxation of liposomes after mixing with tissue clearing solution was monitored at an excitation wavelength of 460 nm using a spectrofluorometer (RF-6000, Shimadzu Corporation, Kyoto, Japan).

### 2.16. Dye Leakage from Liposomes

To evaluate membrane integrity and the importance of pH adjustment, dye leakage from phenolsulfonphthalein (PSP)-containing liposomes was measured. PSP-containing liposomes were prepared using Bangham’s method. The 1,2-Dioleoyl-sn-glycero-3-phosphocholine and cholesterol were dissolved in methanol at a molar ratio of 70:30. Then, a thin lipid film was formed using a rotary evaporator and subsequently desiccated overnight. To obtain PSP-containing liposomes, the thin lipid film was rehydrated in 5 mg/mL PSP (Nacalai Tesque, 26807-92) in carbonate buffer solution (10 mM sodium carbonate, 150 mM sodium chloride, pH 10) at a final lipid concentration of 20 mg/mL. Liposomes were extruded through polycarbonate membrane filters (200-nm pore sizes, 11 times for each pore size) using a commercially available instrument (Mini-Extruder). To remove PSP in the outer phase, a desalting column (PD-10, GE Healthcare Lifesciences, Cheshire, UK) was used. Then, 200 µL of liposome dispersion was mixed with 1 mL tissue clearing solution, and the mixture was incubated for 1 h at 37 °C. Subsequently, 1 mL of the mixture was diluted with 1.5 mL of the carbonate buffer solution and desalted with a PD-10 column. The eluate (500 µL) was diluted with 1 mL of 1 M NaOH, and PSP absorbance at 550 nm was determined using a UV-vis spectrometer (UV-1850, Shimadzu Corporation). For the intact liposome group, liposomes were diluted with the carbonate buffer solution. For the 100% release group, 1% Triton X-100 in the carbonate buffer solution was used as the diluent. The percentage of released PSP was calculated using the following equation.

(1) $$\%\;\mathrm{release}=\left(1-\frac{ABS_{sample}-ABS_{triton}}{ABS_{intact}-ABS_{triton}}\right)\times 100,$$

### 2.17. Supplementary Experiments

Methods for supplementary experiments were described in a supplementary information file.

### 2.18. Data Analysis

Exact *p*-values for each experiment were calculated using R (version 3.5.1). Two-tailed, unpaired *t*-tests or one-way ANOVA followed by Dunnett’s or Tukey’s test were performed to compare experimental groups as indicated in the figure legends. Numerical conversion of fluorescence in images were performed using Fiji (Image J, NIH). Fiji, Zen Black, and Zen Blue (Carl Zeiss Microscopy) software were used for image visualization including scale bar drawing. Zen Blue software was used for maximum intensity projections, average intensity projections, and three-dimensional (3D) renderings. Adobe Photoshop (Adobe Systems Inc., San Jose, CA, USA) was used for inputs of scale information to 3D-rendered images. Microsoft PowerPoint (Microsoft Corporation, Redmond, WA, USA) was used for figure arrangements. Adobe Photoshop was used for resolution adjustments without image resampling.

## 3. Results

### 3.1. Optimization of Seebest Solution

**Figure 1:**
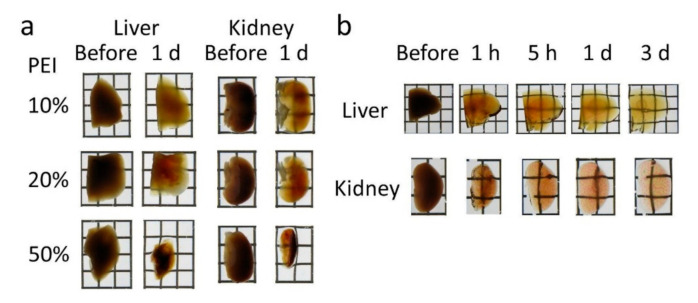

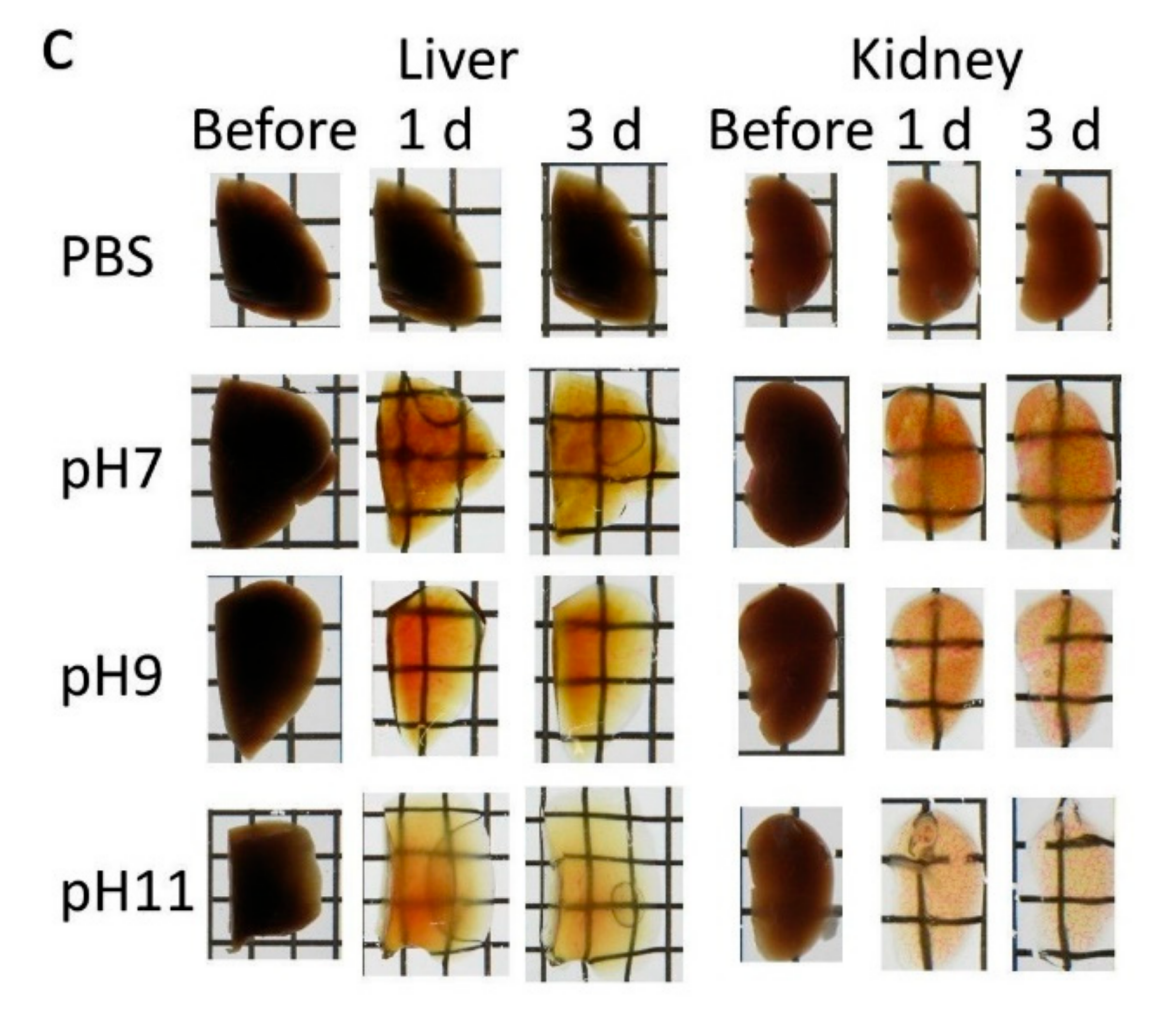
Tissue clearing using Seebest. (**a**) Murine liver and kidney cleared by polyethylenimine (PEI) solutions (molecular weight, MW 1800; 10, 20, and 50 *w*/*v*%). (**b**) Clearing speed of Seebest. DiI-perfused liver and kidney were cleared using Seebest [pH 11, PEI (MW 1800) 20 *w*/*v*% + 8 M urea]. (**c**) The pH dependency of tissue clearing using Seebest. DiI-perfused liver and kidney were cleared using Seebest [pH 7, 9, and 11, PEI (MW 600) 10 *w*/*v*% + PEI (MW 10,000) 10 *w*/*v*% + 8 M urea]. Each lattice was 4 mm wide and 4 mm high. Each image was representative of at least three experiments.

The experimental system employing tissue homogenates has been used as a quantitative screening method [8]. Both the physical appearance and tissue homogenates were used to evaluate the tissue clearing efficiency. PEI itself had a weak tissue clearing effect on tissues and homogenates (Figure 1a and Figure S1, Supplementary Materials). The optical density of each blank homogenate was decreased by increasing the wavelengths. Without PEI, increasing the urea concentration decreased the optical density. Addition of PEI further decreased the optical density, especially at low urea concentrations. There were no obvious differences in optical densities among different urea concentrations with PEI. Conversely, addition of a high concentration of urea to the PEI solution enhanced the tissue clearing efficiency (Figures S2 and S3). Because PEI is a macromolecule, we tested the molecular weight dependency of tissue clearing (Figure S4, Supplementary Materials). Liver and kidney cleared with Seebest containing high molecular weight PEI (molecular weight, MW 10,000) were clearer than those cleared with Seebest containing low molecular weight PEI (MW 600). However, Seebest containing high molecular weight PEI (MW 10,000) shrank the liver and kidney. Next, we found that a combination of low and high molecular weight PEI sufficiently cleared the liver and kidney while maintaining the tissue size. The clearing speed of Seebest was extremely high because 1-h-treated samples were transparent (Figure 1b). Decreasing the pH of Seebest slightly decreased transparency. However, even at neutral pH (pH 7), Seebest sufficiently cleared the liver and kidney (Figure 1c). Tissue size changes were monitored directly over time (Figure S5, Supplementary Materials). Although slight shrinkage was observed at the initial stage after immersion, Seebest pH 8.5 maintained the liver sample size for 3 days. Conversely, Seebest pH 11 induced notable swelling. This may explain the difference in tissue clearing efficiencies, that is, swelling in Seebest pH 11 may lead to more transparency. In terms of the other tissue clearing reagents, *Clear^T2^* shrank liver samples whereas Sca*l*eA2 and Sca*l*eSQ(0) swelled liver samples. Therefore, sample size changes were highly dependent on tissue clearing reagents.

### 3.2. Deep Imaging of Blood Vessels in Various Tissues with Seebest

We performed blood vessel staining with a lipophilic carbocyanine dye, DiI [23], for deep imaging of blood vessels in various tissues with Seebest (Figure S6, Supplementary Materials). DiI was perfused into mice to stain blood vessels. The tissue clearing speed after immersion in Seebest differed for each tissue (Figure S6a, Supplementary Materials). In addition to the liver and kidney, the heart, spleen, and small and large intestines were cleared at 1 day after immersion. At 3 days, the brain, lung, and stomach were efficiently cleared compared with immersion for 1 day. DiI staining visualized different blood vessel structures among the various tissues (Figure S6b, Supplementary Materials). Blood vessels in the brain exhibited a sharp shape, whereas liver blood vessels were blurred because of the fenestrated endothelium. In the kidney, glomerular structures were visualized. In the small intestine, villus structures were visible. Observable depths of DiI signals were dependent on observing conditions, such as lens magnification, WD, and NA. The observable depth for the brain was the deepest under a ×10 dry lens (WD: 5.2 mm; NA: 0.3) at more than 2000 µm. Observable depths for most tissues, except for the lung, were around 1000 µm or more. The shallowest observable depth of the lung was about 400 μm, but we considered this depth sufficient.

There are several lipophilic carbocyanine dyes, including DiO, DiI, and DiD. Next, we stained different structures using multiple carbocyanine dyes. Brain parenchyma was stained by immersing a thick brain slice into DiD solution and then blood vessels and parenchyma were simultaneously visualized (Figure S7, Supplementary Materials).

### 3.3. Comparison with Other Tissue Clearing Methods

To compare the tissue clearing efficiency, we used tissue homogenates (Figure S8, Supplementary Materials). For all tested tissue homogenates, clearing efficiency of *Clear^T2^* was insufficient. Sca*l*eA2 and SQ(0) had moderate clearing efficiencies for all tissue homogenates, whereas CUBIC had the best performance. Seebest and Sca*l*eS4 had sufficient clearing capacities. For liver and kidney homogenates (Figure S8a,b, Supplementary Materials), optical densities of Seebest and Sca*l*eS4 were comparable with those of CUBIC. For brain homogenates (Figure S8c, Supplementary Materials), although the optical densities of Seebest were higher than those of CUBIC, the absolute values were sufficiently small.

**Figure 2:**
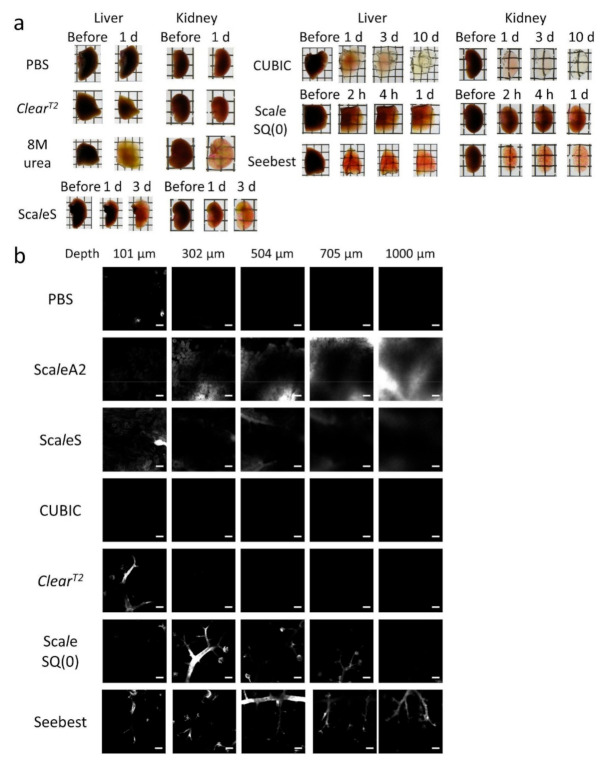
Comparison of tissue clearing methods. (**a**) Murine liver and kidney cleared by various tissue clearing methods. Each lattice was 4 mm wide and 4 mm high. (**b**) Observable depths of blood vessels in the kidney of DiI-perfused mice. Fluorescence images were acquired as a z-stack at 6.714-µm intervals using a confocal microscope with a ×10 EC Plan-Neofluar objective lens. Acquisition settings were as follows: zoom, 1.0; xy scaling, 0.830 µm; spectral emission filters (bandwidth), 547–740 nm; laser wavelength, 543 nm. Seebest pH was 11. Scale bars represent 100 µm. Each image was representative of three experiments.

For liver and kidney tissues (Figure 2a), *Clear^T2^* was also insufficient to clear the tissues. The tissue clearing efficiency of the CUBIC protocol was extremely high, but it required a relatively long time to reach a steady state. Sca*l*eS, Sca*l*eSQ(0), and 8 M urea had similar clearing efficiencies for the liver and kidney. To quantitatively compare the clearing speed, we developed an experimental system using a tissue mimic with a defined thickness in a 96-well plate. The tissue-mimic-on-a-plate comprised a tissue homogenate and gelatin gel, and absorbance of each well was measured over time using a plate reader (Figure S9, Supplementary Materials). The slopes of the curves were calculated based on the initial 30-min data (Table S1) and the slopes were considered to be the speed. The clearing speed of FUnGI was extremely high. That of Seebest pH 11 was slightly slower than that of FUnGI, but the difference was not significant. Those of CUBIC reagents 1 and 2 were moderate, whereas those of Sca*l*eSQ(0) and *Clear^T2^* were significantly slower than that of FUnGI. The clearing efficiency of Seebest for liver and kidney was sufficiently high and clearing speed was very high. DiI-stained blood vessels were observable in the liver and kidney cleared by Seebest. Importantly, the Seebest procedure was convenient because of immersion in a single solution.

The applicability of tissue clearing methods for visualization of blood vessels stained with DiI was compared (Figure 2b). Using detergent-containing methods, Sca*l*eA2, Sca*l*eS, and CUBIC, blood vessels were invisible. Among detergent-free methods—that is—*Clear^T2^*, Sca*l*eSQ(0), and Seebest, Seebest had the best observable depth (>1000 μm) for blood vessels stained with lipophilic dye DiI.

### 3.4. Preservation of Lipid Ultrastructures

**Figure 3:**
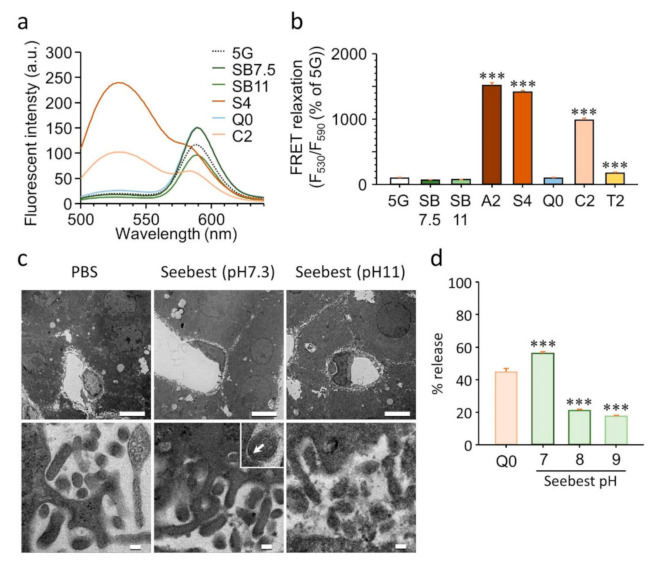
Assessment of lipid structures during tissue clearing. (**a**) Fluorescence spectra of Förster resonance energy transfer (FRET) liposomes in various tissue clearing solutions. (**b**) FRET relaxation of FRET liposomes by breaking lipid membranes. Moreover, 5G, 5% glucose; SB 7.5 and 11, Seebest pH 7.5 and 11; A2, Sca*l*eA2; S4, Sca*l*eS4; Q0, Sca*l*eSQ(0); C2, CUBIC reagent 2; T2, *Clear^T2^*. Each bar represents the mean + S.D. of three experiments. Statistical comparison was performed using Dunnett’s test. The 5G group served as the control. *** *p* < 0.001 vs. control group. (**c**) Transmission electron microscopy. Liver specimens were subjected to transmission electron microscopy 1 day after incubation in Seebest. Each image was representative of three experiments. Scale bars represent 5 µm (upper panels) and 0.1 µm (lower panels). White arrow in the enlarged region of the lower panel of Seebest pH 7.3 indicates the lipid bilayer of the plasma membrane. (**d**) Release of dyes from phenolsulfonphthalein (PSP)-liposomes induced by tissue clearing solutions. Q0, Sca*l*eSQ(0). Each bar represents the mean + S.D. of three experiments. Statistical comparison was performed using Dunnett’s test. The Q0 group served as the control. *** *p* < 0.001 vs. control group.

To assess the preservability of lipid membrane integrity, we first applied in vitro FRET, which detect membrane lysis. Integrities of FRET liposomes treated with tissue clearing solutions were monitored using FRET relaxation (Figure 3a,b). If membrane lysis occurs, FRET relaxation is observed by disintegration of donor and acceptor fluorescent molecules, that is, *N*-(7-nitro-2-1,3-benzoxadiazol-4-yl) (excitation, 460 nm; emission, 530 nm) and *N*-(lissamine rhodamine B sulfonyl) (excitation, 556 nm; emission, 590 nm) moieties, respectively. A high FRET relaxation value represents membrane lysis. Detergent-containing solutions, Sca*l*eA2, Sca*l*eS4, and CUBIC-2, significantly relaxed the FRET, which indicates a break of the lipid membrane. Although *Clear^T2^* does not contain a detergent, *Clear^T2^* slightly relaxed the FRET and affected membrane integrity. However, Seebest (pH 7.5 and 11) and Sca*l*eSQ(0) maintained FRET efficiency, which suggests preservation of lipid membranes.

It has been reported that Sca*l*eS protocols maintain ultrastructures whereas CUBIC does not [11]. Therefore, we used transmission electron microscopy to determine whether Seebest maintained ultrastructures (Figure 3c). Upper panels in Figure 3c show sinusoids and hepatocytes, and lower panels show microvilli of hepatocytes. Seebest pH 11 partly disarranged the lipid ultrastructure. This disarrangement might be attributed to swelling of the sample (Figure S5, Supplementary Materials). Conversely, Seebest pH 7.3 maintained lipid ultrastructures because the lipid bilayer was visible.

To evaluate membrane integrity using the quantitative experimental system of dye leakage, we used PSP-containing liposomes as a model (Figure 3d). The proportions of ionic and non-ionic forms for PSP depend on pH. Above the pKa of PSP (pKa1 = 7.39; pKa2 = 7.99), the ionic form is predominant. Permeation of the ionic form through the membrane should be restricted. Therefore, an intact membrane may suppress leakage of the ionic form of PSP. In Seebest above pH 8, 20% of PSP leaked from liposomes. Therefore, almost 80% of PSP remained in the liposomes. Conversely, only 40% of PSP remained in liposomes incubated with Seebest pH 7. Membrane-preserving tissue clearing solution Sca*l*eSQ(0) released approximately 40% of PSP from liposomes. Therefore, not only membrane integrity, but also pH adjustment were important factors to suppress dye leakage.

### 3.5. Importance of Adjusting pH for Visualization of Biological Events and States

Numerous fluorescent dyes detect biological events and states. Many dyes require membrane integrity to maintain signals. Membrane solubilization often results in dye loss. Moreover, most dyes have pH-dependent fluorescence. Seebest maintained membrane integrity and was pH adjustable. Therefore, we expected the applicability of Seebest to visualize biological events and states using fluorescent dyes. As biological events and states, we focused on the spatial disposition of an unfixable fluorescent drug Dox, and oxidative stress using various low molecular weight dyes.

**Figure 4:**
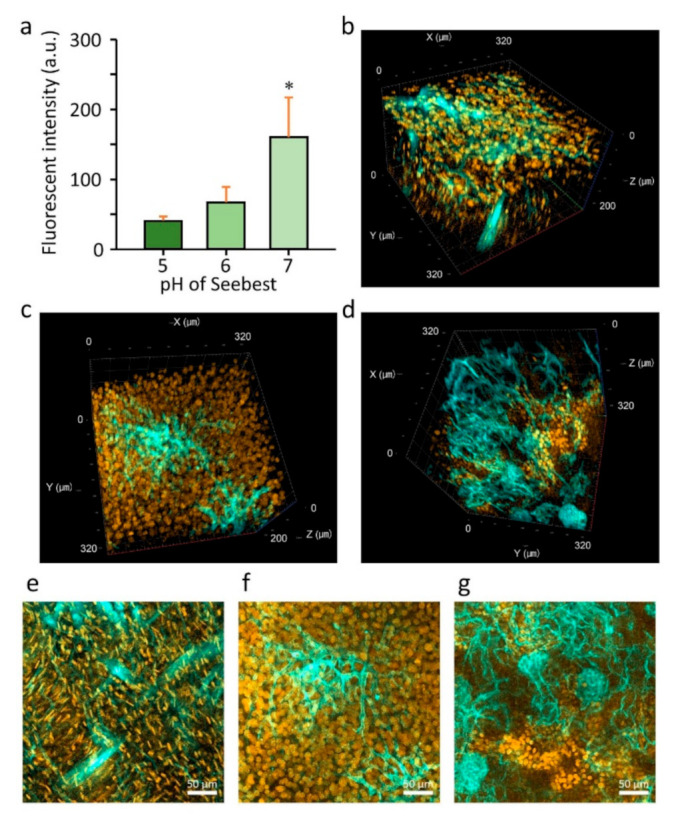
Visualization of the Dox spatial distribution. (**a**) Dox release during tissue clearing in Seebest (pH 5–7). Each bar represents the mean + S.D. of three experiments. Statistical comparison was performed using Dunnett’s test. * *p* < 0.05 vs. pH 5 group. (**b**–**d**) Dox (orange) and DiD-stained blood vessel (cyan) visualization in (**b**) the heart, (**c**) liver and (**d**) kidney. Maximum intensity projections of (**b**–**d**) were also presented in (**e**–**g**), respectively. Scale bars represent 50 µm. Seebest pH was 6. Fluorescence images were acquired as a z-stack at 1.222-µm intervals using a confocal microscope with a ×25 LD LCI Plan-Apochromat objective lens. Acquisition settings were as follows: zoom, 1.0; xy scaling, 0.664 µm; spectral emission filters (bandwidth), 527–600 nm and 639–758 nm; laser wavelengths, 488 and 633 nm for Doxorubicin (DOX) and DiD, respectively. Each image was representative of three experiments.

To assess the importance of adjusting pH, we examined the pH dependency of the fluorescent spectra of dyes (Figure S10, Supplementary Materials). DiI had little pH dependency (Figure S10a, Supplementary Materials). Incubation with Seebest pH 7–9 or CUBIC did not affect the fluorescent intensity of green fluorescent protein ZsGreen1, while incubation with Seebest pH > 10 decreased the stability of ZsGreen1 (Figure S10b, Supplementary Materials). Conversely, Dox had great pH dependency and acidic pH maximized the fluorescence (Figure S10c, Supplementary Materials). For CellROX Deep Red, a fixable reagent that detects general ROS, the fluorescent spectra depended on the pH of Seebest (Figure S10d, Supplementary Materials). At pH 8–8.5, the fluorescent peak reached the maximum. Acidic pH suppressed Dox release from the fixed liver during incubation in Seebest (Figure 4a). Next, we observed the spatial disposition of Dox in the heart, liver, and kidney (Figure 4b–d). Considering the balance between tissue clarity and Dox leakage, we chose Seebest pH 6 for this experiment. Dox fluorescence was located in the nuclei of these tissues, which indicates successful observation of Dox.

**Figure 5:**
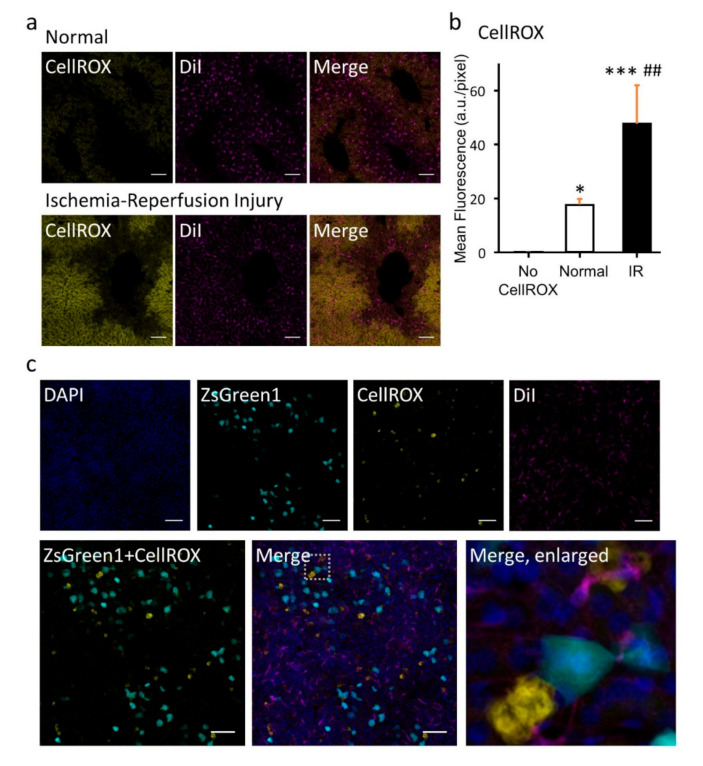
ROS visualization. (**a**) ROS (yellow) were detected using CellROX Deep Red reagent in normal and ischemia/reperfusion-injured livers. Magenta signals represent DiI-stained sinusoids. (**b**) Numerical conversion of CellROX fluorescence in untreated (no CellROX), normal, and ischemia/reperfusion (IR)-injured livers, calculated by the average of z-stack images (0–40 µm). Each bar represents the mean + S.D. of four regions. Statistical comparison was performed using Tukey’s test. * *p* < 0.05, *** *p* < 0.001 vs. no CellROX group, ## *p* < 0.01 vs. normal group. (**c**) Relationship between gene expression and ROS after hydrodynamics-based transfection to the liver. Blue, DAPI-stained nuclei. Cyan, gene expression of ZsGreen1. Yellow, ROS detected using CellROX Deep Red. Magenta, DiI-stained sinusoids. Fluorescence images were acquired as a z-stack at (**a**) 1.000-µm and (**c**) 0.637-µm intervals using a confocal microscope with a ×40 LD C-Apochromat objective lens. Acquisition settings were as follows: zoom, 1.0; xy scaling, 0.415 µm; spectral emission filters (bandwidth), 409–484, 494–543, 553–631, and 648–726 nm; laser wavelengths, 405, 488, 543, and 633 nm for DAPI, ZsGreen1, DiI, and CellROX Deep Red, respectively. Each panel represents average intensity projection of (**a**) 40.00 µm and (**c**) 38.23 µm in thickness, with a 4 × 4 tile observation. Scale bars represent 100 µm. Each image was representative of three experiments.

We chose Seebest pH 8.5 to visualize ROS in liver ischemia-reperfusion (IR) injury (Figure 5a,b). It was obvious that the generated ROS in IR injury was observable with Seebest, whereas signals of the basal ROS level in normal mice were weak. There were interregional differences in ROS generation (Figure 5a). During hypoxia in the ischemia phase, labile Fe^2+^ ions are released from iron proteins such as cytochrome P450 in the liver, which catalyze Fenton’s reaction to produce hydroxyl radicals from H_2_O_2_. These hydroxyl radicals are severe ROS that oxidize lipids, proteins, and sugars. We used unfixable SiRhoNox-1 [24] to detect labile Fe^2+^ ions in IR injury (Figure S11a and Video S1, Supplementary Materials). SiRhoNox-1 is not a chelator of Fe^2+^, but is converted into a fluorescent form by labile Fe^2+^ ions [24]. In this experiment, we used Seebest pH 7.5. Punctate signals of labile Fe^2+^ ions were observed outside of nuclei. It has been reported that hypoxia-induced release of labile Fe(II) can be detected by SiRhoNox-1 that localizes in the endoplasmic reticulum (ER). Our results regarding punctate signals of labile Fe^2+^ ions were consistent with the previous reports [24,25]. Furthermore, we simultaneously observed labile Fe^2+^ ions and ROS to analyze the relationship (Figure S11b, Supplementary Materials). To prevent the fluorescence overlap, we used unfixable HMRhoNox-M [25] instead of SiRhoNox-1. HMRhoNox-M is also converted into a fluorescent form by labile Fe^2+^ ions [25]. Fluorescent signals of HMRhoNox-M were undetectable at pH 8.5, whereas CellROX Deep Red signals were observable at pH 8.0 and 8.5. These results clearly indicated the necessity for pH adjustment. At pH 8.0, signals of HMRhoNox-M for labile Fe^2+^ ions were substantially merged with CellROX signals for general ROS generation. However, there were distinguishable CellROX signals from HMRhoNox-M, which suggests that generation of ROS other than hydroxyl radials in IR injury was detected. To prove the importance of both membrane preservation and pH-adjustment, in vitro experiments using human hepatoma HepG2 cells were performed (Figure S12, Supplementary Materials). Although immersion of HepG2 cells with Seebest pH 8.0 slightly changed the disposition of HMRhoNox-M, HMRhoNox-M was detectable using Seebest pH 8.0, while Seebest pH 7.5 and 8.5 reduced the HMRhoNox-M signals. Using Seebest, autofluorescence was reduced compared with PBS. Addition of detergent Triton X-100 to Seebest pH 8.0 diminished the HMRhoNox-M signals. In case of Sca*l*eSQ(0), HMRhoNox-M signals were hardly detectable, probably due to the swelling as shown in DAPI signals.

Hydrodynamics-based transfection to the liver [26,27] is a highly efficient in vivo gene transfer method. Hydrodynamics-based transfection generates ROS, and it is related to high gene expression. Therefore, we analyzed the relative spatial distribution of gene expression and ROS generation (Figure 5c and Video S2). Interestingly, ROS levels in gene expression-positive cells were not high, but regions of ROS generation were certainly near gene expression-positive cells. Similar results were obtained using unfixable CellROX Orange instead of fixable CellROX Deep Red (Figure S13, Supplementary Materials). Seebest was slightly effective compared with Sca*l*eSQ(0).

### 3.6. Immunohistochemical Staining

Compatibility with immunohistochemical staining is important for tissue clearing methods. Seebest without detergents maintained lipid membranes. Therefore, Seebest itself should be incompatible with immunohistochemical staining. After three-dimensional observation, however, we might be able to observe immunofluorescence if Seebest preserved the antigenicity of cleared tissues. To check the preservation of antigenicity at 1 day after immersion in Seebest, a cleared liver was washed with phosphate-buffered saline (PBS) (−) and subjected to immunohistochemical staining (Figure S14a, Supplementary Materials). Tubulin staining with an anti-tubulin antibody clearly visualized microtubule structures in the liver. To prove preservation of tubulin antigenicity for longer time, we utilized a cell culture experiment which guaranteed complete contact of all cells with Seebest. Tubulin was also visible after 3-day-immersion of HepG2 cells with Seebest (Figure S14b, Supplementary Materials); therefore, immersion with Seebest preserved tubulin antigenicity for at least 3 days.

### 3.7. Improvement of the Multicolor Deep Imaging Potential of the Seebest Solution

**Figure 6:**
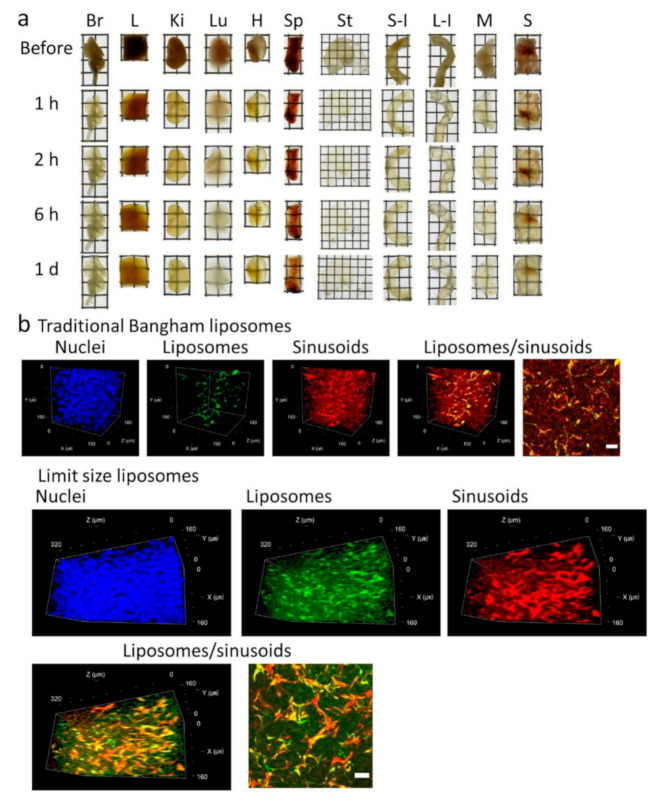
Seebest-PP performance. Seebest-PP solution comprising 8 M urea and 20 *w*/*v*% propylene oxide-denatured PEI was used. (**a**) Before and at 1, 2, and 6 h, and 1 day after immersion in Seebest, tissues were observed. Br, brain; L, liver; Ki, kidney; Lu, lung; H, heart; Sp, spleen; St, stomach; S-I, small intestine; L-I, large intestine; M, muscle; S, skin. Each lattice was 4 mm wide and 4 mm high. (**b**) Visualization of the spatial distribution of liposomes in the liver. DiO-labelled traditional Bangham liposomes or limit size liposomes were intravenously injected into mice. DiI was also intravenously injected at 1.5 h after liposome injection. Thirty minutes after DiI injection, the liver specimens were subjected to confocal microscopy. Fluorescence images were acquired as a z-stack at 1.090-µm intervals using a confocal microscope with a ×25 LD LCI Plan-Apochromat objective lens. Acquisition settings were as follows: zoom, 2.0; xy scaling, 0.664 µm; spectral emission filters (bandwidth), 409–484, 494–543, and 550–670 nm; laser wavelengths, 405, 488, and 543 nm for DAPI, DiO, and DiI, respectively. Maximum intensity projections of liposomes/sinusoids were also presented. Scale bars represent 20 µm. Each image was representative of three experiments.

During preparation and storage, the Seebest solution acquired a slightly yellowish color. The spectrum of the Seebest solution indicated light absorbance, especially at low wavelengths (Figure S15a, Supplementary Materials). This result explained the yellowish color. The light absorbance at low wavelength might restrict imaging of violet-blue fluorescence, such as DAPI for nuclear observation, and decrease the multicolor deep imaging potential of the Seebest solution. The light absorbance of the Seebest solution may be due to the presence of primary and/or secondary amines. These amino groups may react with each other and produce azo compounds, which generally have a yellow color. Here, propylene oxide-denatured PEI has neither primary nor secondary amines. Seebest solution with propylene oxide-denatured PEI (Seebest-PP) decreased the absorbance of the Seebest solution with normal PEI at a low wavelength (Figure S15b, Supplementary Materials). Seebest-PP also cleared various tissues including muscle and skin (Figure 6a). Furthermore, the observable depth for DAPI in Seebest-PP was approximately twice as deep as that in normal Seebest (Figure S15c, Supplementary Materials). We applied Seebest-PP to visualize the spatial distribution of liposomes in the liver (Figure 6b). The spatial distribution of traditional Bangham liposomes and limit size liposomes [28] were compared. Bangham liposomes of 202.6 ± 1.6 nm in diameter (polydispersity index, 0.333 ± 0.011, n = 3) were distributed mainly to endothelial and Kupffer cells. In addition to such distribution characteristics, limit size liposomes of 60.9 ± 1.2 nm in diameter (polydispersity index, 0.392 ± 0.013, n = 3) were distributed in part to hepatocytes, which indicates penetration through the fenestrated endothelium. 

## 4. Discussion

We succeeded in development of the novel tissue optical solution Seebest. Blood vessels stained with a lipophilic carbocyanine dye were deeply observable with Seebest (Figure 2 and Figure S6, Supplementary Materials). Seebest was applicable to multiple tissues including the brain, liver, kidney, spleen, heart, lung, stomach, and small and large intestines (Figure S6, Supplementary Materials). Tissue clearing efficiency of Seebest was dependent on pH, but Seebest substantially cleared tissues even at neutral pH (Figure 1c). The clearing procedure of Seebest was simple, that is, immersion in only one solution. The clearing speed of Seebest was very high (Figure 1b, Figure S9 and Table S1, Supplementary Materials). Importantly, Seebest around neutral pH only slightly changed the sample size during clearing (Figure 1 and Figure S5, Supplementary Materials) and preserved lipid ultrastructures at the electron microscopy level (Figure 3). We applied Seebest to visualization of the relative spatial distribution of gene expression and oxidative stress with nuclear and sinusoidal fluorescence staining (Figure 5). Thus, Seebest provides pH adjustable, simple, rapid and effective tissue optical clearing while preserving lipid ultrastructures for multicolor deep bioimaging.

The most important advancement in tissue clearing using Seebest was the pH adjustment. Numerous fluorescent dyes and proteins have been developed. Although most fluorescent dyes and proteins are sensitive to pH in terms of fluorescence spectrum, intensity, and stability, little attention has previously focused on pH during tissue clearing. Simultaneously, Seebest preserved plasma membranes, which enabled us to stain lipidic structures with fluorescent dyes. Considering the pH adjustment and preserved plasma membranes, we expected that Seebest could retain small ionic fluorescent molecules in cells during tissue clearing (Figure S16, Supplementary Materials). Conventional tissue optical clearing methods with detergents, including CLARITY, CUBIC, Sca*l*eS and UBasM, affect the plasma membrane. Consequently, it is impossible to retain free fluorescent dyes in cells. Although there are several detergent-free tissue clearing methods, including *Clear^T2^*, SeeDB, and Sca*l*eSQ(0), pH during tissue clearing is not adjusted in these protocols. Therefore, the equilibrium should shift from ionic to molecular forms because of the loss of the molecular form of probes. Sample size changes during immersion in a clearing solution might be another important factor to prevent dye leakage. The pH of Sca*l*eSQ(0) is above the pKa of PSP, but Sca*l*eSQ(0) did not prevent PSP leakage from liposomes (Figure 3d). This might be attributed to the increase in sample size during immersion in Sca*l*eSQ(0) (Figure S5, Supplementary Materials). To date, tissue clearing requires fixation of fluorescent molecules. Seebest could minimize the loss of small fluorescent molecules during tissue clearing by adjusting the pH to maximize the proportion of the ionic form. Tissue clearing using Seebest was extremely quick. Thus, we succeeded in observing the small molecule Dox before its loss (Figure 4). However, the suppression of dye leakage was incomplete even in Seebest (Figure 3d and Figure S12, Supplementary Materials). This limitation might be attributed to the slight changes in sample size during immersion in Seebest (Figure S5, Supplementary Materials).

Seebest’s tissue clearing mechanism is unclear. Essentially, tissues are opaque mainly because of light scattering by various components with different refractive indices. Tissue clearing methods generally homogenize these refractive indices [29]. Several strategies can be used to adjust the refractive index, such as dehydration, lipid removal, and use of materials with high refractive indices such as sugars and urea. PEI itself had a partial clearing effect (Figure 1a). This effect might be due to dehydration because the molecular weight of PEI is too high to penetrate cells. The refractive index of the PEI solution was not as high as that of the urea solution (Table 1). Obviously, the combination of PEI and urea synergistically enhanced the tissue clearing capacity (Figure 1 and Figure 2). Urea penetration might be an important factor for tissue clearing using Seebest. Except for *Clear^T2^*, absolute clearing capacities for tissue homogenates did not vary significantly among tissue clearing solutions (Figure S8, Supplementary Materials). Additionally, the refractive indices of these solutions were comparable (Table 1). Thus, not only the refractive index of the solution, but also other factors, such as penetration speed should be involved in effective tissue clearing. Observable depths for DiI-stained blood vessels depended on tissues. Although the clearing capacity for the brain homogenate was comparable to those for liver and kidney homogenates (Figure S2, Supplementary Materials), the observable depth of the brain was much deeper. This result might be due to the contents of heme iron and elastic fibers. In the case of the lung, air in the trachea and alveoli might interfere with tissue clearing.

Usability of 400–700 nm wavelengths is preferable for multicolor deep imaging using confocal microscopy. Although Seebest employing PEI was compatible to four-color imaging (Figure 5c), it had slight difficulty in observing DAPI with the 405-nm excitation laser. This should be attributed to the light absorbance at the low wavelength (Figure S15a, Supplementary Materials). The cause of light absorbance at a low wavelength might be the production of impurities that were probably azo compounds. To reduce the reactivity of PEI, we used propylene oxide-denatured PEI. Use of propylene oxide-denatured PEI to prepare the Seebest solution (Seebest-PP) successfully reduced light absorbance at low wavelengths (Figure S15b, Supplementary Materials). This improved the observable depth of DAPI (Figure S15c, Supplementary Materials) and indicates an increase in the multicolor deep imaging potential. Using Seebest-PP, we observed spatial distributions of liposomes in relation to blood vessels (Figure 6b). We also successfully elucidated different spatial distribution characteristics between traditional Bangham and limit size liposomes. Thus, Seebest-PP may be useful to visualize the spatial distribution of lipidic drug delivery system (DDS) carriers. About evaluation of spatial distribution in DDS fields, we have reported several applications using lipid-preserving tissue clearing methods *Clear^T2^* and Sca*l*eSQ(0) [30,31,32,33,34,35]. In these reports, we have evaluated positional relationship of spatial distribution of gene expression with blood vessels or tissue surface stained with lipidic carbocyanine dyes DiI. Seebest and Seebest-PP are thought to be applicable to such evaluation. Moreover, Seebest-PP would maximize multicolor deep imaging potency to simultaneously evaluate positional relationship of gene expression and/or lipidic carriers (such as liposomes and lipid nanoparticles) with biological structures (such as blood vessels, tissue surface and elastic fibers), oxidative stress and so on. Moreover, we have developed the quantification method of DDS carrier concentration in tissue homogenates using tissue clearing solutions [36]. We are currently optimizing the composition of Seebest-PP to determine the concentration of lipidic DDS carriers in tissues. Thus, Seebest solution would realize not only multicolor deep imaging of spatial distribution, but also easy quantification of various DDS carriers [37].

Clearing using Seebest did not reduce antigenicity. Of course, Seebest was incompatible with three-dimensional immunohistochemistry because of the preserved plasma membrane. The permeabilization procedure during immunohistochemistry destroys lipid structures. Therefore, preservation of lipid structures and immunohistochemistry contradict each other. However, sequential observations before and after immunohistochemistry would enable us to perform ultra-multicolor deep imaging, namely three-dimensional correlative twice light microscopy. At least one color should serve as a reference color for the first observation. After the first observation, the other colors are removed by solubilization and/or photobleaching and staining with antibodies is performed. In the second observation, the corresponding region with the first observation can be observed according to the pattern of the reference color. We also plan to apply Seebest to clinical biopsies such as cancers and non-alcoholic steatohepatitis.

## 5. Conclusions

In this study, we demonstrated the compatibility of Seebest with lipophilic carbocyanine dyes, ROS probes, fluorescent protein ZsGreen1, DAPI nuclear staining, and small molecular fluorescent drug Dox. In terms of applicability, Seebest would be suitable to not only biological events and states, but also evaluation of DDS carriers. Furthermore, we will apply Seebest to imaging of collagen structures, autophagy, and cell death in tissues. Because of preserved lipid ultrastructures, Seebest will also be applicable to correlative light and electron microscopy. Additionally, clearing using Seebest preserved antigenicity. The Seebest solution provides pH-adjustable, rapid, sufficient tissue clearing while preserving lipid ultrastructures, which is suitable for multicolor deep imaging. Hence, Seebest solution facilitates the elucidation of biological events and states in tissues.

## Figures and Tables

**Table 1:** Properties of aqueous solution-based tissue clearing methods.

Solution	pH	Detergent	Refractive Index	Solution Exchange	Clearing Capacity ^(1)^	Time Required
Seebest	Variously adjusted (5–12)	No	1.438–1.442	Not necessary	++++	1 h–1 d
(PEI 20%)	Variously adjusted (5–12)	No	1.367–1.371	-	+	-
(Urea 8 M)	Not buffered (7.9)	No	1.399	-	++	-
Sca*l*eA2	Not buffered (8.4)	Yes	1.383	Optional	++	2 w
Sca*l*eS	Not buffered (8.1, S4)	Yes	1.425 (S4)	Necessary (5 solutions)	+++	3 d
Sca*l*eSQ(0)	Not buffered (8.6)	No	1.444	Optional	+++	2 h–1 d
CUBIC	Uses original alkaline pH (9.7, reagent 2)	Yes	1.454 (reagent 2)	Necessary (3 solutions)	+++++	1–2 w
*Clear^T2^*	Not buffered (6.4)	No	1.413	Necessary (3 solutions)	+	1 d

^(1)^ +++++ extremely strong; ++++ very strong; +++ strong; ++ moderate; + weak.

## Supplementary Materials

The following are available online at https://www.mdpi.com/1999-4923/12/11/1070/s1, Figure S1: Changes in absorbance of tissue homogenates induced by PEI, Figure S2: Changes in absorbance of tissue homogenates induced by urea solutions with or without 20 *w*/*v*% PEI, Figure S3: Effect of addition of 20 *w*/*v*% PEI to the urea solution on tissue clarity, Figure S4: Effect of molecular weight of PEI on tissue clarity, Figure S5: Tissue size change during tissue clearing, Figure S6: Clearing various tissues in Seebest, Figure S7: Simultaneous visualization of blood vessels and parenchyma in the brain, Figure S8: Comparison of the absorbance of tissue homogenates in tissue clearing solution, Figure S9: Quantitative comparison of clearing speed, Figure S10: pH dependency of fluorescence, Figure S11: Visualization of labile Fe^2+^ ions in the liver, Figure S12: Importance of membrane preservation and pH-adjustment to detect low molecular fluorescent dye, Figure S13: Visualization of ROS with CellROX Orange in the liver, Figure S14: Compatibility with immunohistochemistry after immersion in Seebest, Figure S15: Improvement of the multicolor deep imaging potential of Seebest using propylene oxide-denatured PEI, Figure S16: Schematic representation of minimizing the release of small fluorescent molecules from cells, Table S1: Comparison of clearing speeds of various tissue clearing solutions using a tissue mimic on a plate, Video S1: Rotatory image for visualization of labile Fe^2+^ ions in the liver of ischemia/reperfusion-injured mice, Video S2: Rotatory image for relationship between transgene expression and ROS after hydrodynamics-based transfection to the liver.
