# Supplementary material for: A pH-Adjustable Tissue Clearing Solution That Preserves Lipid Ultrastructures: Suitable Tissue Clearing Method for DDS Evaluation
Source: Pharmaceutics. 2020 Nov 9;12(11):1070. doi: 10.3390/pharmaceutics12111070 (PMC7698078; doi:10.3390/pharmaceutics12111070)
Supplement: Supplementary file 1 [file pharmaceutics-12-01070-s001.zip › Pharmaceutics-Supplementary-Information done-done.pdf]

Supplementary Information

A pH-adjustable tissue clearing solution that preserves lipid ultrastructures: Suitable tissue clearing method for DDS evaluation

Shintaro Fumoto, Eriko Kinoshita, Keisuke Ohta, Kei-ichiro Nakamura, Tasuku Hirayama, Hideko Nagasawa, Die Hu, Kazuya Okami, Riku Kato, Shojiro Shimokawa, Naho Ohira, Koyo Nishimura, Hirotaka Miyamoto, Takashi Tanaka, Shigeru Kawakami and Koyo Nishida

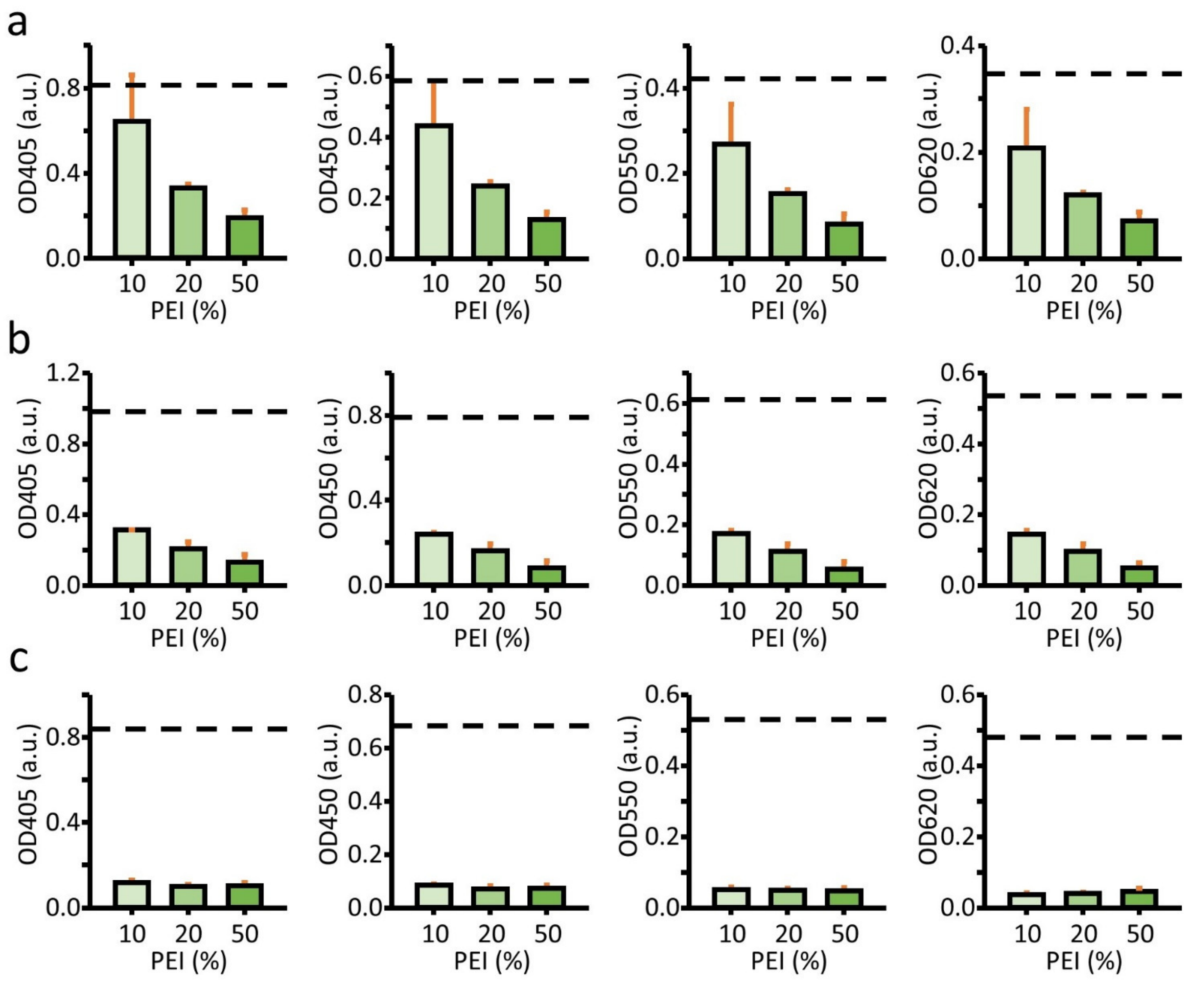

Figure S1. Changes in absorbance of tissue homogenates induced by PEI. (**a**) Liver, (**b**) kidney, and (**c**) brain homogenates. Each panel indicates absorbance of samples at different wavelengths as indicated in vertical axis. Dashed lines represent the absorbance of blank homogenates diluted in PBS (−). Bars represent the mean + S.D. of three experiments.

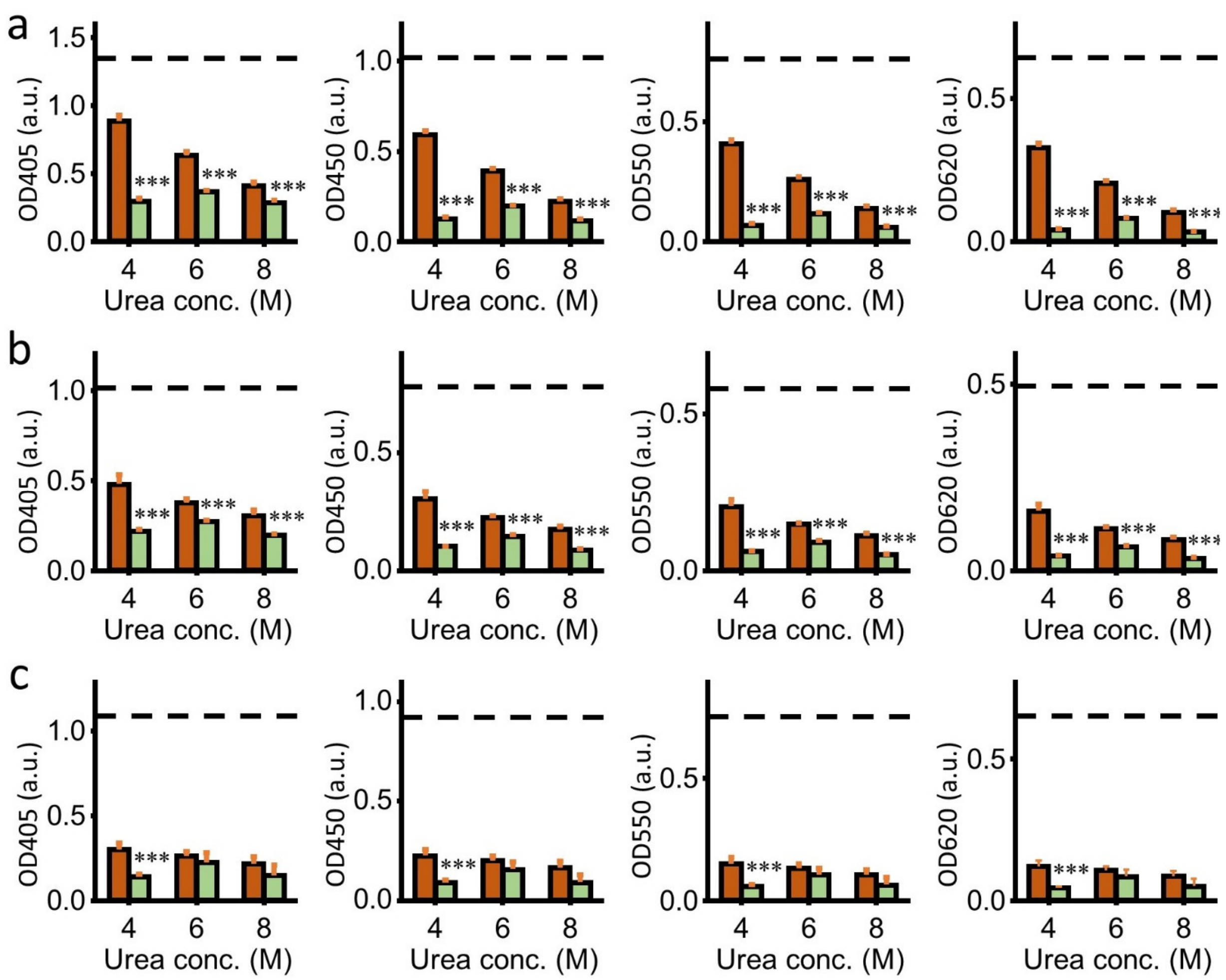

Figure S2. Changes in absorbance of tissue homogenates induced by urea solutions with or without 20 *w*/*v*% PEI. (**a**) Liver, (**b**) kidney, and (**c**) brain homogenates. Each panel indicates absorbance of samples at different wavelengths as indicated in vertical axis. Dashed lines represent the absorbance of blank homogenates diluted in PBS (−). Orange (without PEI) and green bars (with PEI) represent the mean + S.D. of three experiments. Statistical comparisons were performed by two-tailed unpaired t-tests. ***$p < 0.001$.

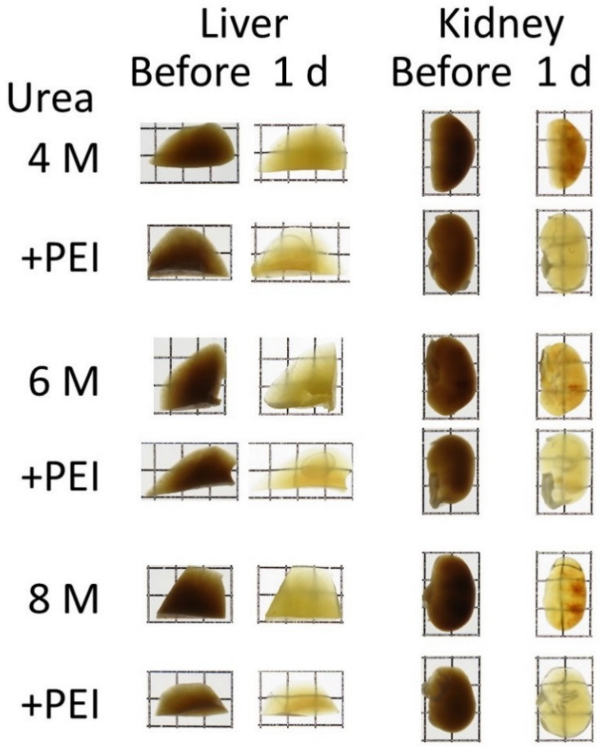

Figure S3. Effect of addition of 20 *w*/*v*% PEI to the urea solution on tissue clarity**.** Before and at 1 day after immersion in each solution, the liver and kidney were observed. Each lattice was 4 mm wide and 4 mm high. Each image was representative of three experiments.

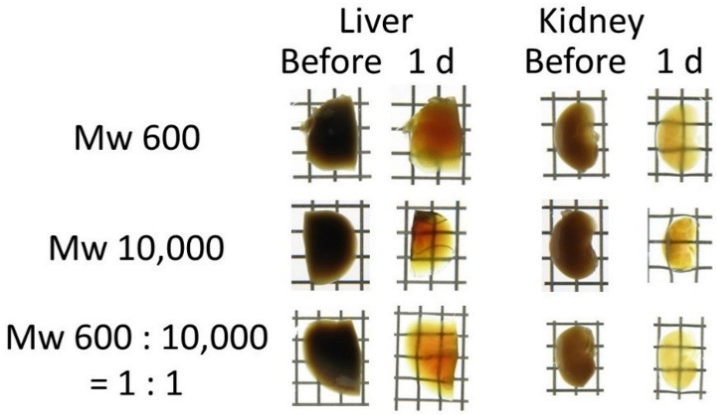

Figure S4. Effect of molecular weight of PEI on tissue clarity**.** Seebest solutions comprising 8 M urea and 20 *w*/*v*% PEI (MW 600, 10,000, and 600:10,000 = 1:1, pH 11) were used. Before and at 1 day after immersion in each solution, the liver and kidney were observed. Each lattice was 4 mm wide and 4 mm high. Each image was representative of three experiments.

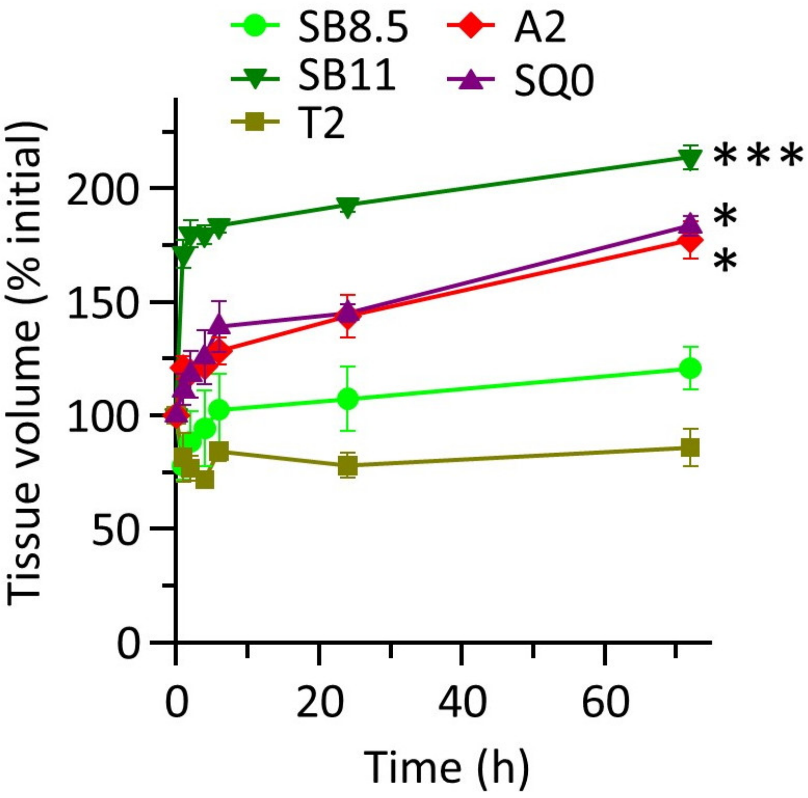

Figure S5. Tissue size change during tissue clearing. Seebest solutions comprising 8 M urea and 20 *w*/*v*% PEI (600:10,000 = 1:1, pH 8.5 and 11) were used. Tissue size changes after immersion in tissue clearing solutions were monitored over time. SB, Seebest; A2, Sca*l*eA2; SQ0, Sca*l*eSQ(0); T2, *Clear*^{T2}. Each symbol represents the mean ± S.E. of three experiments. Statistical comparisons were performed using repeated measures ANOVA followed by Dunnett's test. *$p < 0.05$ and ***$p < 0.001$ vs. Seebest pH 8.5 group.

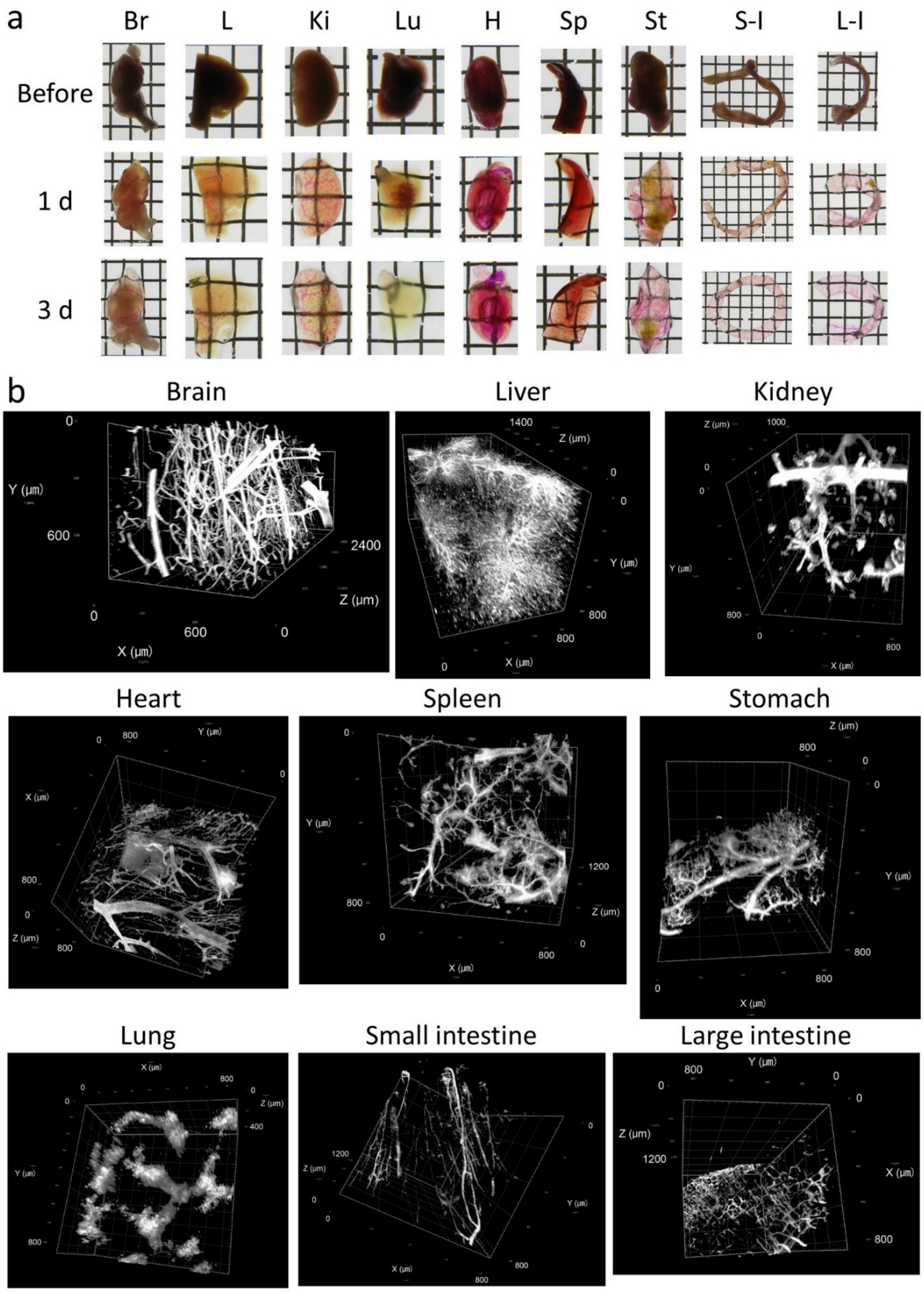

Figure S6. Clearing various tissues in Seebest. Seebest solution comprising 8 M urea and 20 w/v % PEI (600:10,000 = 1:1, pH 11) was used. (**a**) Before and at 1 and 3 days after immersion in Seebest, DiI-perfused tissues were observed. Br, brain; L, liver; Ki, kidney; Lu, lung; H, heart; Sp, spleen; St, stomach; S-I, small intestine; L-I, large intestine. Each lattice was 4 mm wide and 4 mm high. (**b**) Visualization of DiI-stained blood vessels in various tissues cleared by Seebest. Fluorescence images were acquired as a z-stack at 6.714-μm intervals using a confocal microscope with a ×10 EC Plan-

Neofluar objective lens. Acquisition settings were as follows: zoom, 1.0; xy scaling, 0.830 µm; spectral emission filters (bandwidth), 547–670 nm; laser wavelength, 543 nm. Each image was representative of three experiments.

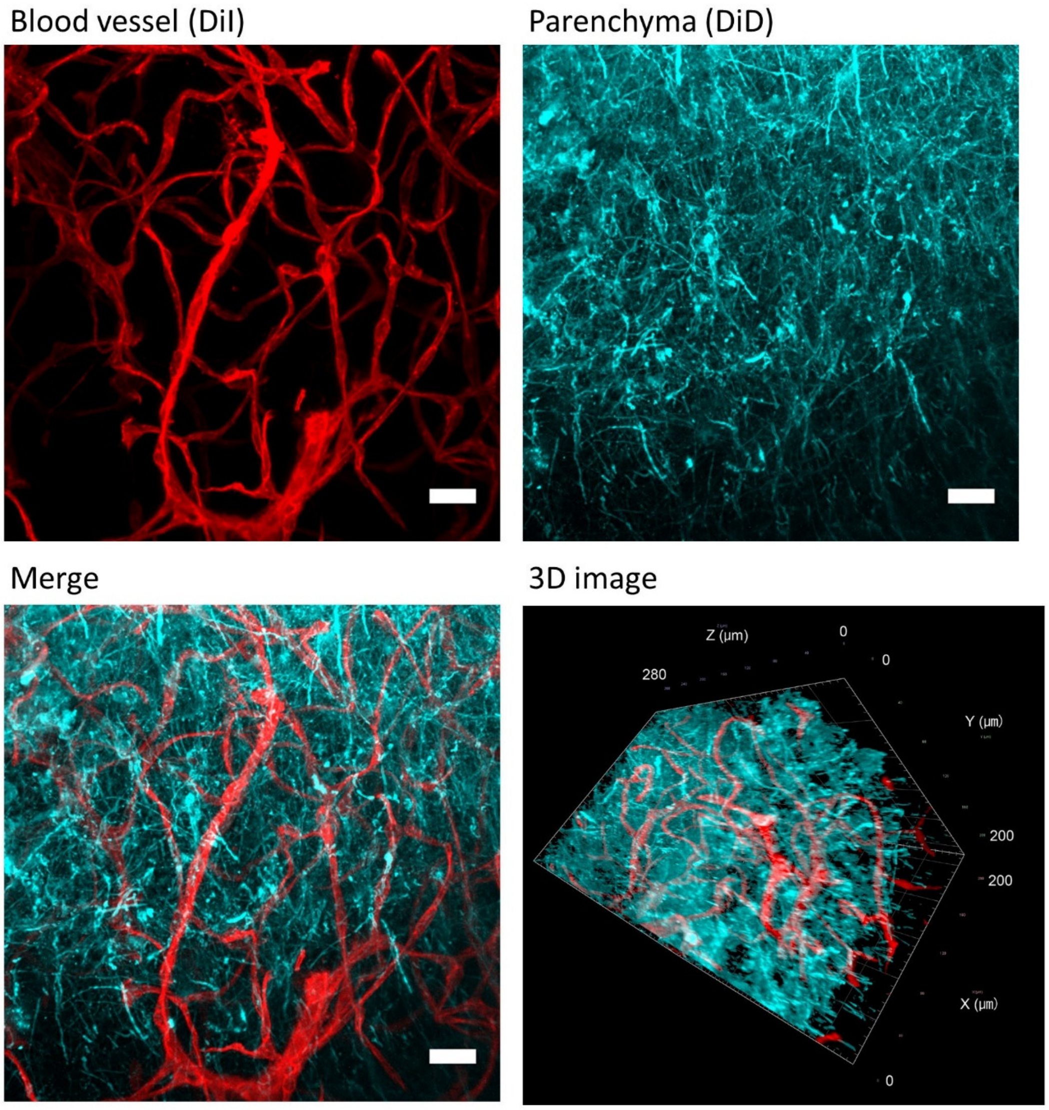

Figure S7. Simultaneous visualization of blood vessels and parenchyma in the brain. After DiI perfusion and fixation, a thick brain slice was immersed in DiD solution, cleared with Seebest (pH 11), and observed by confocal microscopy. Fluorescence images were acquired as a z-stack at 0.700-µm intervals using a confocal microscope with a ×25 LD LCI Plan-Apochromat objective lens. Acquisition settings were as follows: zoom, 1.0; xy scaling, 0.415 µm; spectral emission filters (bandwidth), 560–589 and 637–758 nm; laser wavelengths, 543 and 633 nm for DiI and DiD, respectively. Each panel represents the maximum intensity projection of 312.75 µm in thickness. Each image was representative of three experiments.

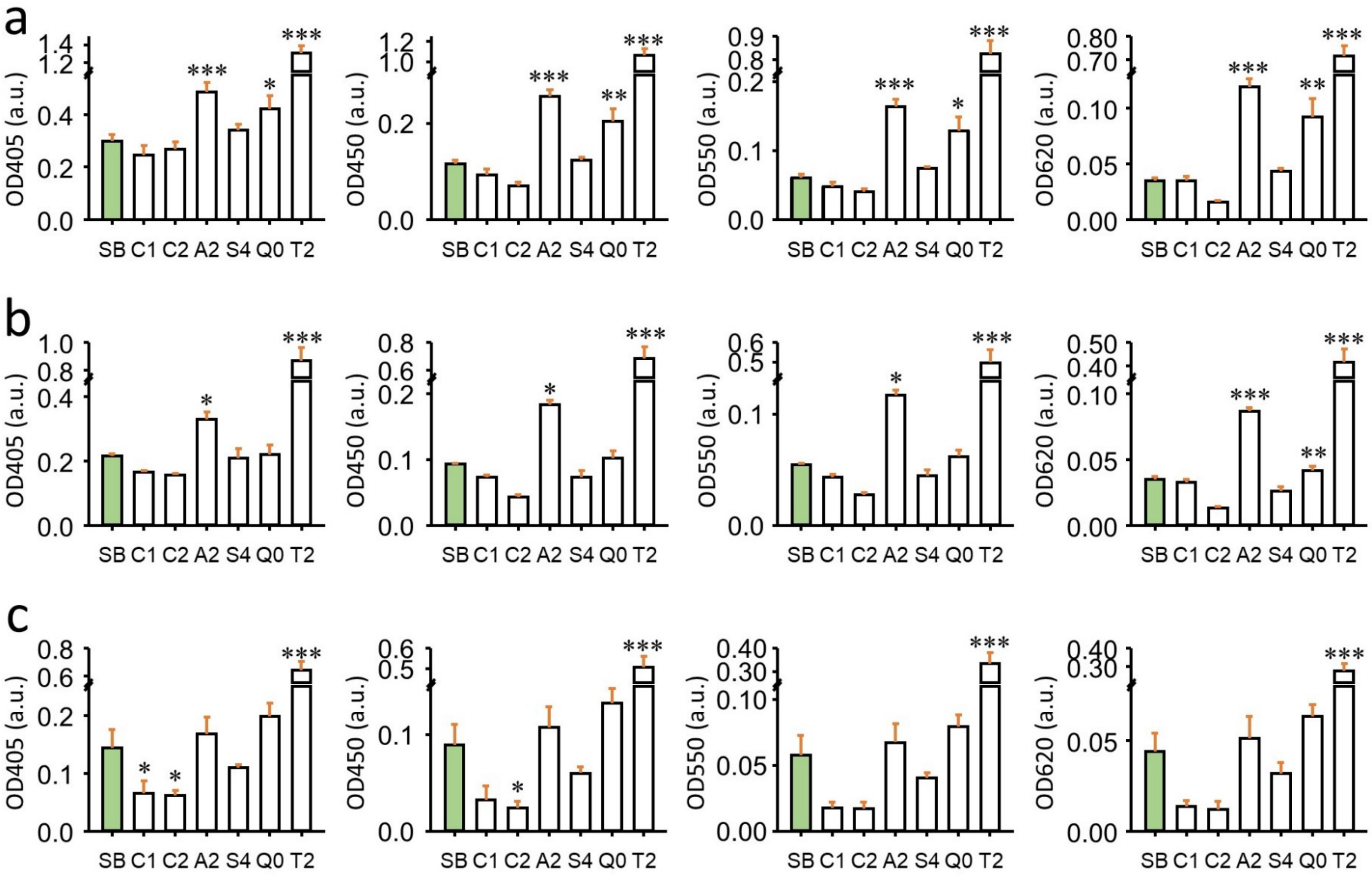

Figure S8. Comparison of the absorbance of tissue homogenates in tissue clearing solution**.** (**a**) Liver, (**b**) kidney, and (**c**) brain homogenates. Each panel indicates absorbance of samples at different wavelengths as indicated in vertical axis. SB, Seebest; C1, CUBIC reagent 1; C2, CUBIC reagent 2; A2, Sca*l*eA2; S4, Sca*l*eS4; Q0, Sca*l*eSQ(0); T2, *Clear*[T2]. Each bar represents the mean + S.D. of three experiments. Statistical comparisons were performed using Dunnett's test. *$p < 0.05$, **$p < 0.01$, and ***$p < 0.001$ vs. Seebest group.

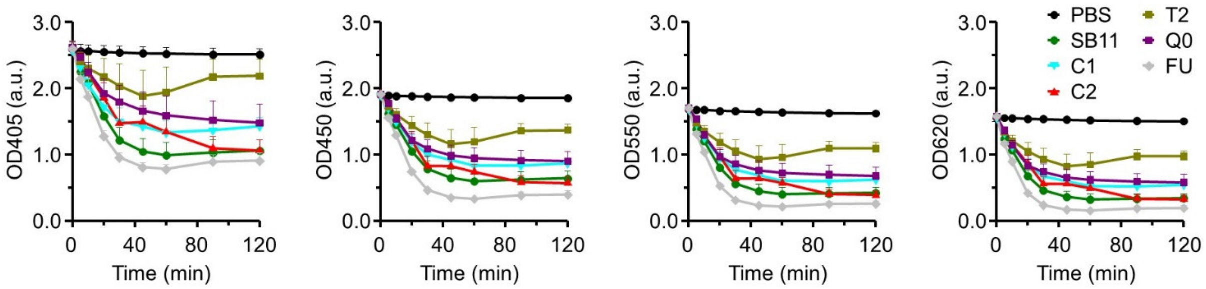

Figure S9. Quantitative comparison of clearing speed. Absorbances of the tissue mimic at 405, 450, 550, and 620 nm were monitored over time. SB11, Seebest pH 11; C1, CUBIC reagent 1; C2, CUBIC reagent 2; T2, *Clear*^{T2}; Q0, Sca*l*eSQ(0); FU, FUnGI. Each symbol represents the mean + S.E. of six experiments.

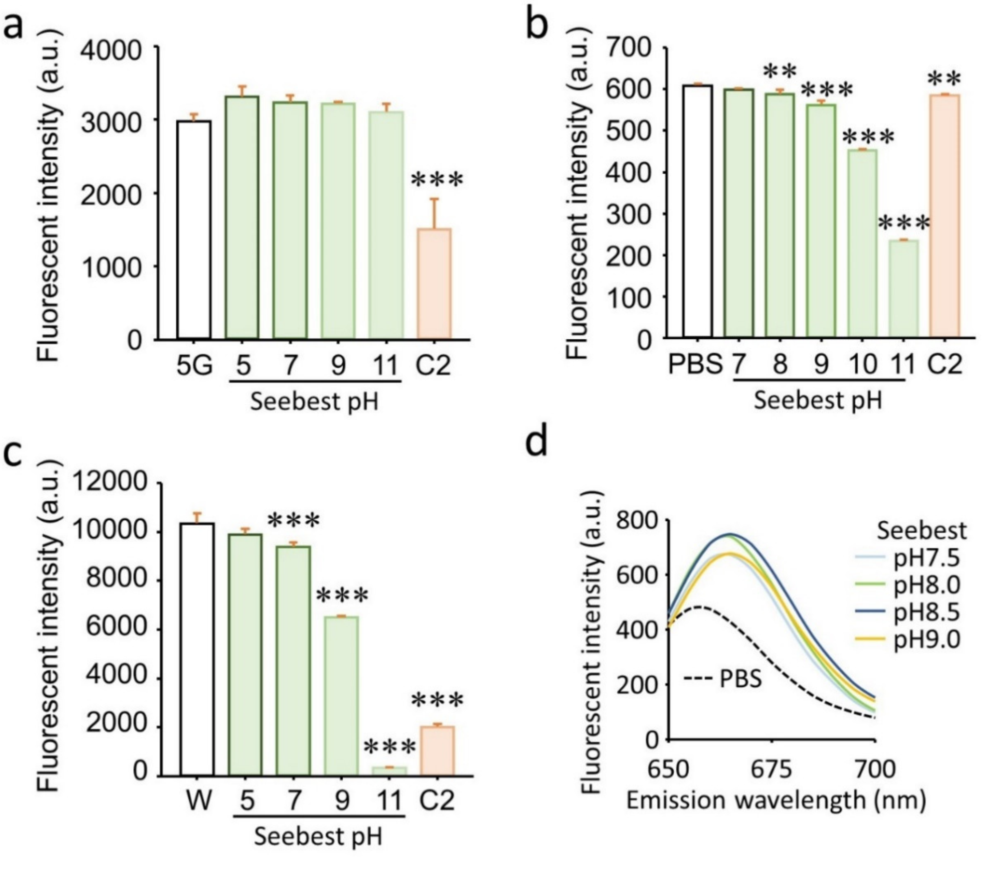

Figure S10. pH dependency of fluorescence**.** (**a**) Fluorescent intensity of DiI liposomes in tissue clearing solutions. 5G, 5% glucose. C2, CUBIC-2. Excitation and emission wavelengths, 543 and 565 nm, respectively. Each bar represents the mean + S.D. of three experiments. Statistical comparison was performed using Dunnett's test. ***$p < 0.001$ vs. 5G group. (**b**) Fluorescent intensity of ZsGreen1 in tissue clearing solution. After incubation for 1 day in tissue clearing solutions, fluorescent intensities were measured. C2, CUBIC-2. Excitation and emission wavelengths, 488 and 510 nm, respectively. Each bar represents the mean + S.D. of three experiments. Statistical comparison was

performed using Dunnett's test. **$p < 0.01$, ***$p < 0.001$ vs. PBS group. (**c**) Fluorescent intensity of Dox in tissue clearing solutions. W, water. C2, CUBIC-2. Excitation and emission wavelengths, 488 nm and 595 nm, respectively. Each bar represents the mean + S.D. of three experiments. Statistical comparison was performed using Dunnett's test. ***$p < 0.001$ vs. W group. (**d**) CellROX Deep Red fluorescence spectra in various Seebest pHs. CellROX Deep Red was activated by incubation in 2 mM H_2O_2 for 1 h at 37 °C and mixed with Seebest. The fluorescence spectrum was then measured. Excitation wavelength, 625 nm.

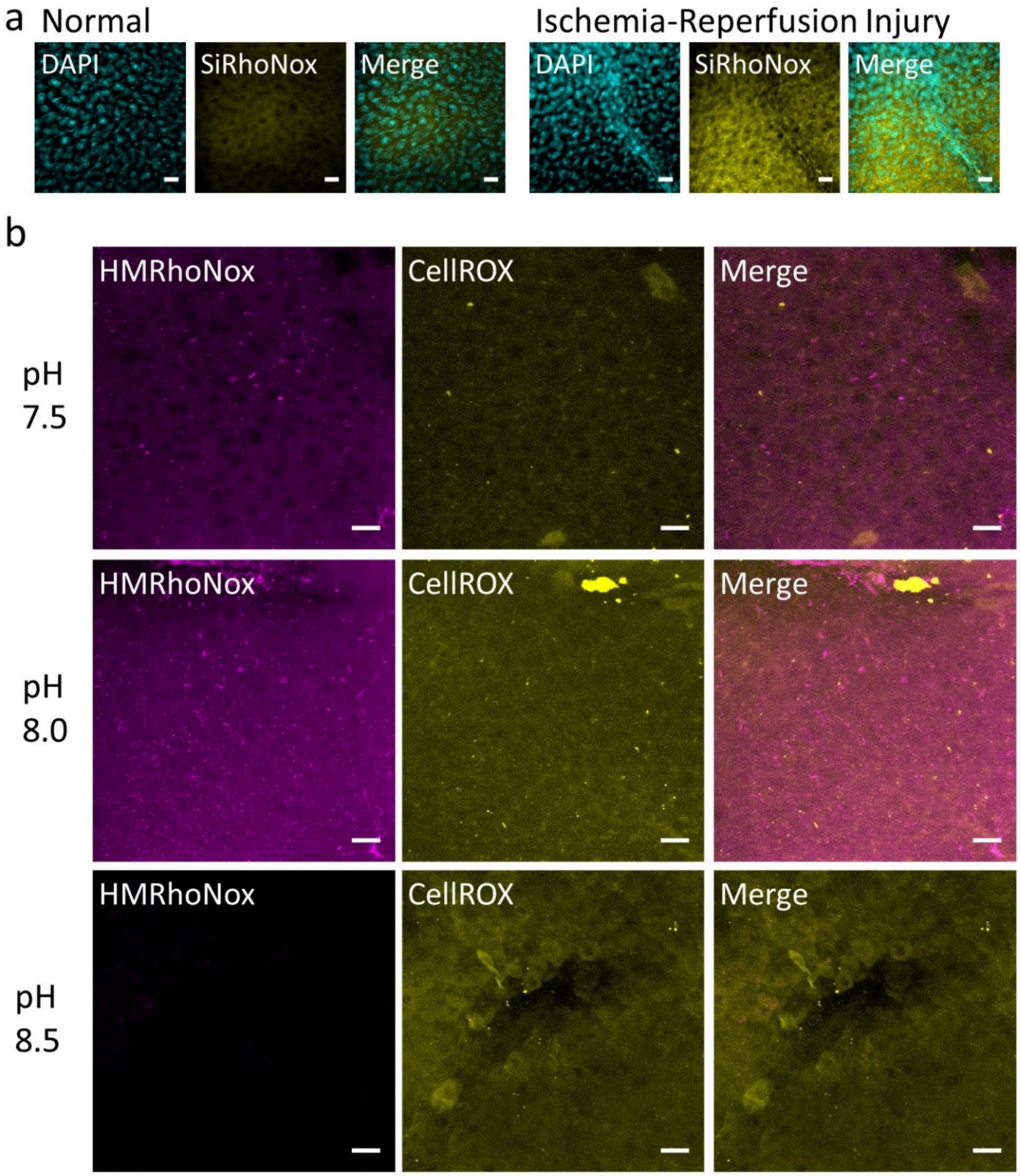

Figure S11. Visualization of labile Fe^{2+} ions in the liver. (**a**) Labile Fe^{2+} ions were detected by SiRhoNox-1 in normal and ischemia/reperfusion-injured murine livers. Seebest pH was 7.5. Cyan, DAPI. Yellow, SiRhoNox-1. (**b**) Simultaneous visualization of general ROS and labile Fe2+ ions using CellROX Deep Red and HMRhoNox-M in ischemia/reperfusion-injured murine liver. Seebest solutions with pH 7.5, 8.0, and 8.5 were used for comparison. Magenta, HMRhoNox-M, Yellow, CellROX Deep Red. Fluorescence images were acquired as a z-stack at (a) 0.688-µm and (b) 0.699-µm intervals using a confocal microscope with a ×40 LD C-Apochromat objective lens. Acquisition settings were as follows: zoom, 1.0; xy scaling, 0.415 µm; spectral emission filters (bandwidth), (a) 409–585 and 637–758 nm for DAPI and SiRhoNox-1, and (b) 413–527, 548–589, and 647–695 nm for DAPI, HMRhoNox-M, and CellROX Deep Red, respectively; laser wavelengths 405, 543, 633, and 633 nm for DAPI, HMRhoNox-M, SiRhoNox-1, and CellROX Deep Red, respectively. Each panel represents (a) the average intensity projection of 102.54 µm and (b) the maximum intensity projection of 101.35 µm in thickness. Scale bars represent 20 µm. Each image was representative of three experiments.

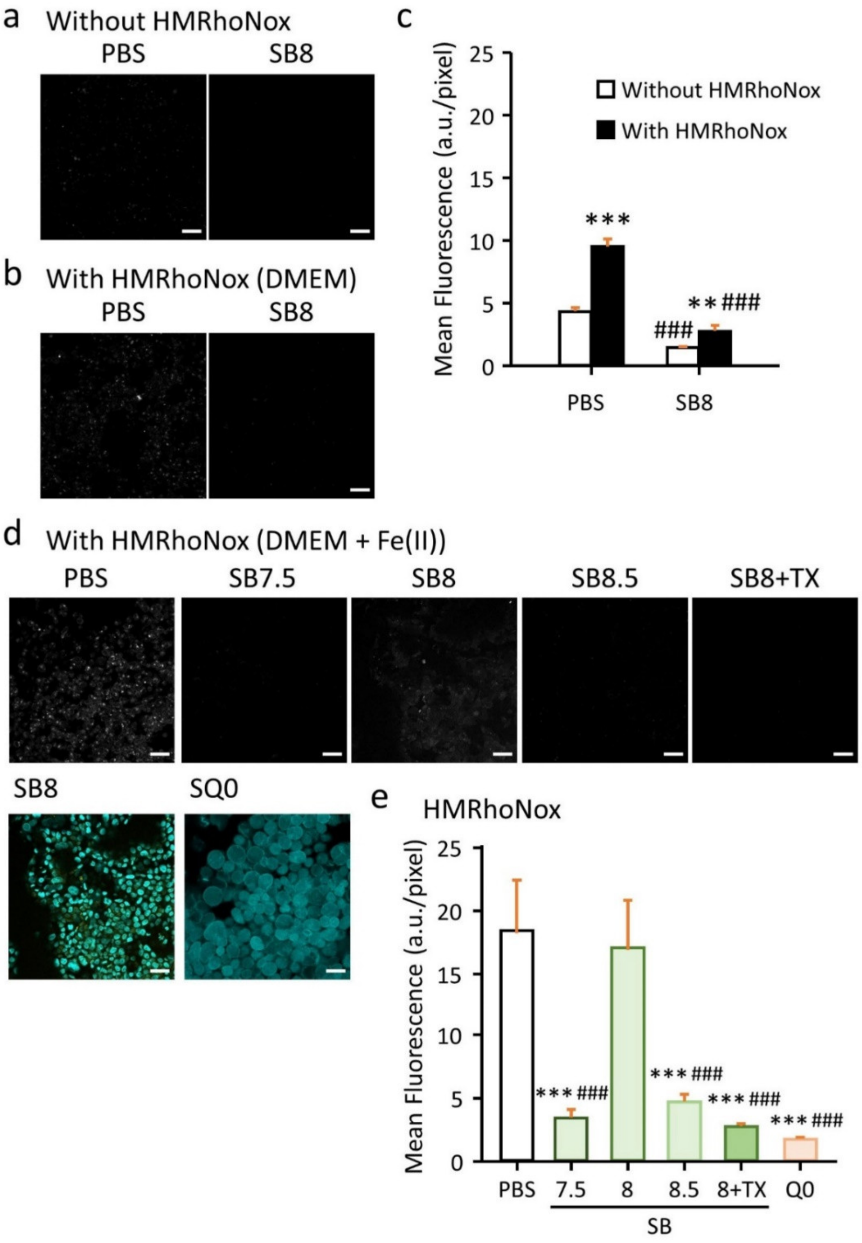

Figure S12. Importance of membrane preservation and pH-adjustment to detect low molecular fluorescent dye. Labile Fe^{2+} ions were detected by HMRhoNox-M in unfixed HepG2 cells treated with ferrous ammonium sulphate (Fe(II)). (**a**) Cells without HMRhoNox-M treatment. (**b**) Cells without Fe(II) treatment. (**c**) Numerical conversion of HMRhoNox-M fluorescence in panel (a) and (b) images. Each bar represents the mean + S.D. of four experiments. Statistical comparison was performed using Tukey's test. ***p* < 0.01, ****p* < 0.001 vs. without HMRhoNox-M group, ###*p* < 0.001 vs. PBS group. (d) Seebest solutions with pH 7.5, 8.0, 8.5, 8.0 plus Triton X-100 (TX, 0.2%), and Sca*l*eSQ(0) were used for comparison. Upper panels, HMRhoNox-M signals. Lower panels, cyan, DAPI; yellow, HMRhoNox-M signals. (e) Numerical conversion of HMRhoNox-M fluorescence in panel (d) images. Each bar represents the mean + S.D. of four experiments. Statistical comparison was performed using Tukey's test. ****p* < 0.001 vs. PBS group, ###*p* < 0.001 vs. Seebest pH 8 group. Fluorescence images were acquired using a confocal microscope with a ×20 Plan-Apochromat objective lens. Acquisition settings were as follows: zoom, 1.0; xy scaling, 0.415 μm; spectral emission filters (bandwidth), 409–521 and 550–670 nm for DAPI and HMRhoNox-M, respectively; laser wavelengths 405 and 543 for DAPI and HMRhoNox-M, respectively. Scale bars represent 50 μm.

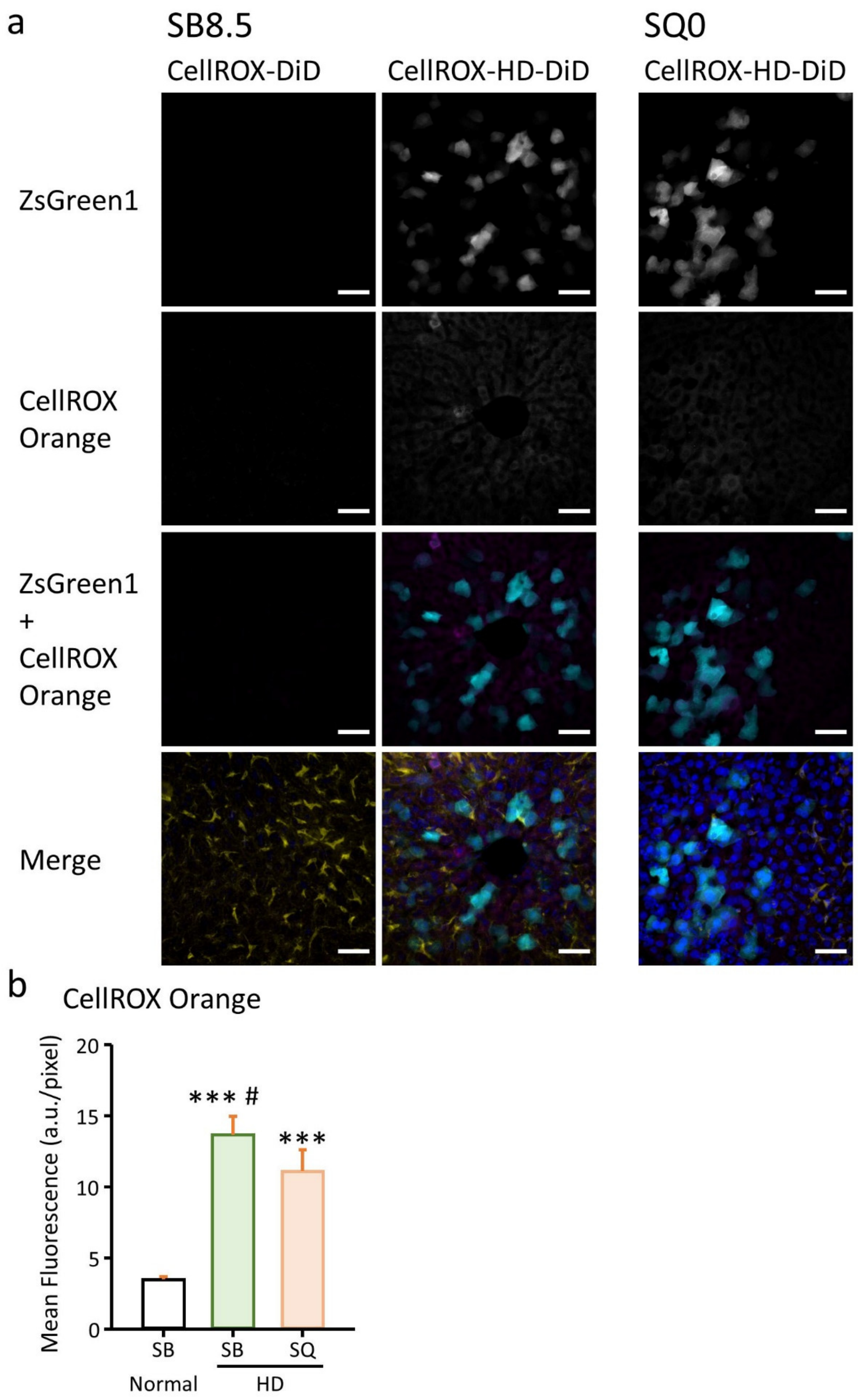

Figure S13. Visualization of ROS with CellROX Orange in the liver. (**a**) Relationship between gene expression and ROS after hydrodynamics-based transfection to the liver. Seebest solutions with 8.5 and Sca*l*eSQ(0) were used for comparison. Blue, DAPI-stained nuclei. Cyan, gene expression of ZsGreen1. Magenta, ROS detected using CellROX Orange. Yellow, DiD-stained sinusoids. (**b**) Numerical conversion of CellROX fluorescence in panel (a), calculated by the average of z-stack

images (0-51.11 μm). Each bar represents the mean + S.D. of four regions. Statistical comparison was performed using Tukey's test. ****p* < 0.001 vs. normal group, #*p* < 0.05 vs. Sca*l*eSQ(0) group. Fluorescence images were acquired as a z-stack at 1.247-μm interval using a confocal microscope with a ×25 LD LCI Plan-Apochromat objective lens. Acquisition settings were as follows: zoom, 1.0; xy scaling, 0.664 μm; spectral emission filters (bandwidth), 409–484, 494–543, 553–631, and 648–726 nm; laser wavelengths, 405, 488, 543, and 633 nm for DAPI, ZsGreen1, CellROX Orange, and DiD, respectively. Each panel represents average intensity projection of 51.11 μm in thickness. Scale bars represent 50 μm. Each image was representative of three experiments.

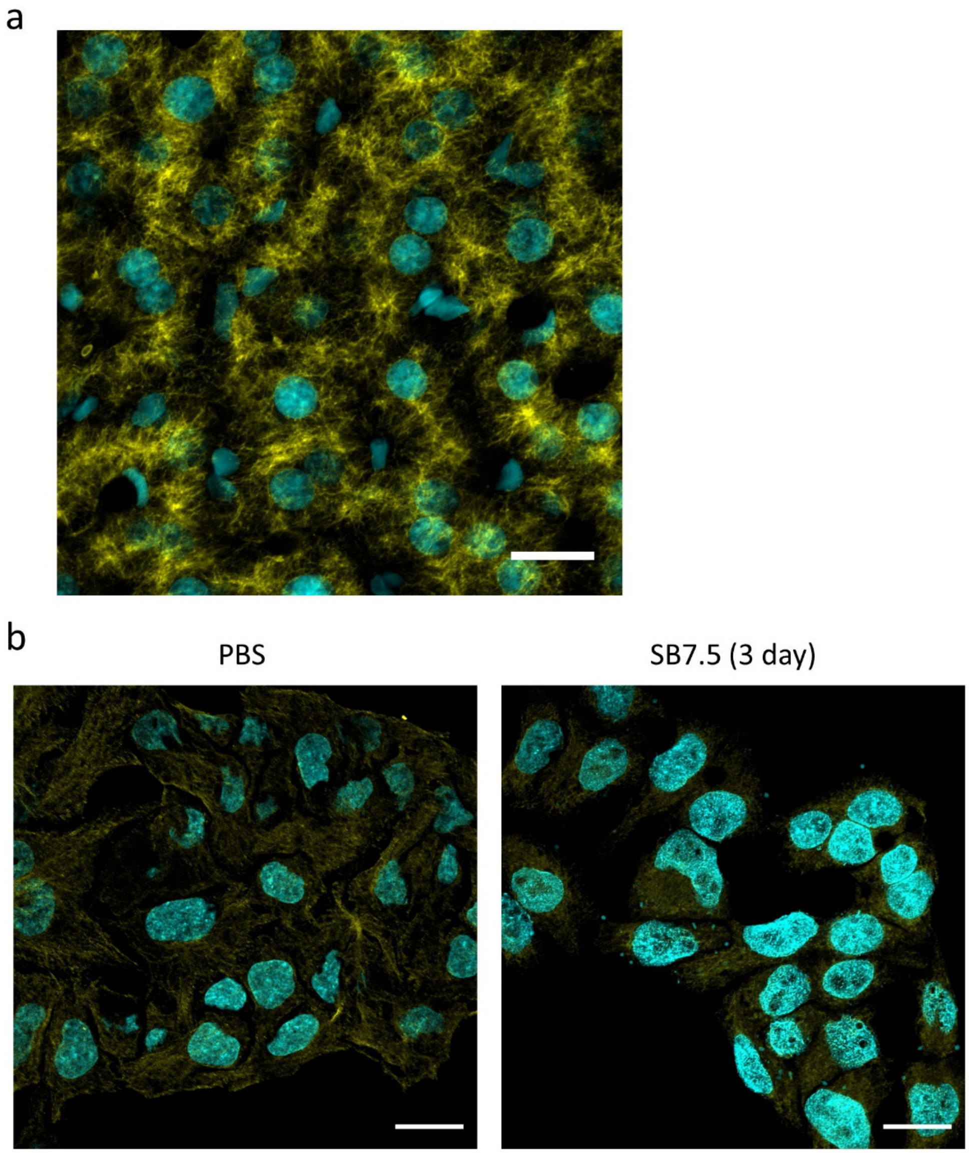

Figure S14. Compatibility with immunohistochemistry after immersion in Seebest**.** (**a**) A fixed liver specimen and (**b**) fixed HepG2 cells were immersed in Seebest (pH 7.5) for (a) 1 day and (b) 3 days at 37°C, washed with PBS (−) for 1 day, and then subjected to immunohistochemistry. Cyan, DAPI. Yellow, Hilyte Fluor 647-labelled anti-tubulin antibody. Fluorescence images were acquired as a z-stack at (a) 0.200-μm and (b) 0.300-μm intervals using a confocal microscope with a ×63 Plan-Apochromat objective lens. Acquisition settings were as follows: zoom, 1.0; xy scaling, 0.132 μm; spectral emission filters (bandwidth), 415–557 and 637–757 nm; laser wavelengths, 405 and 633 nm

for DAPI and Hilyte Fluor 647, respectively. The panel represents the average intensity projection of (a) 14.00 μm and (b) 3 μm in thickness. Scale bars represent 20 μm. Each image was representative of three experiments.

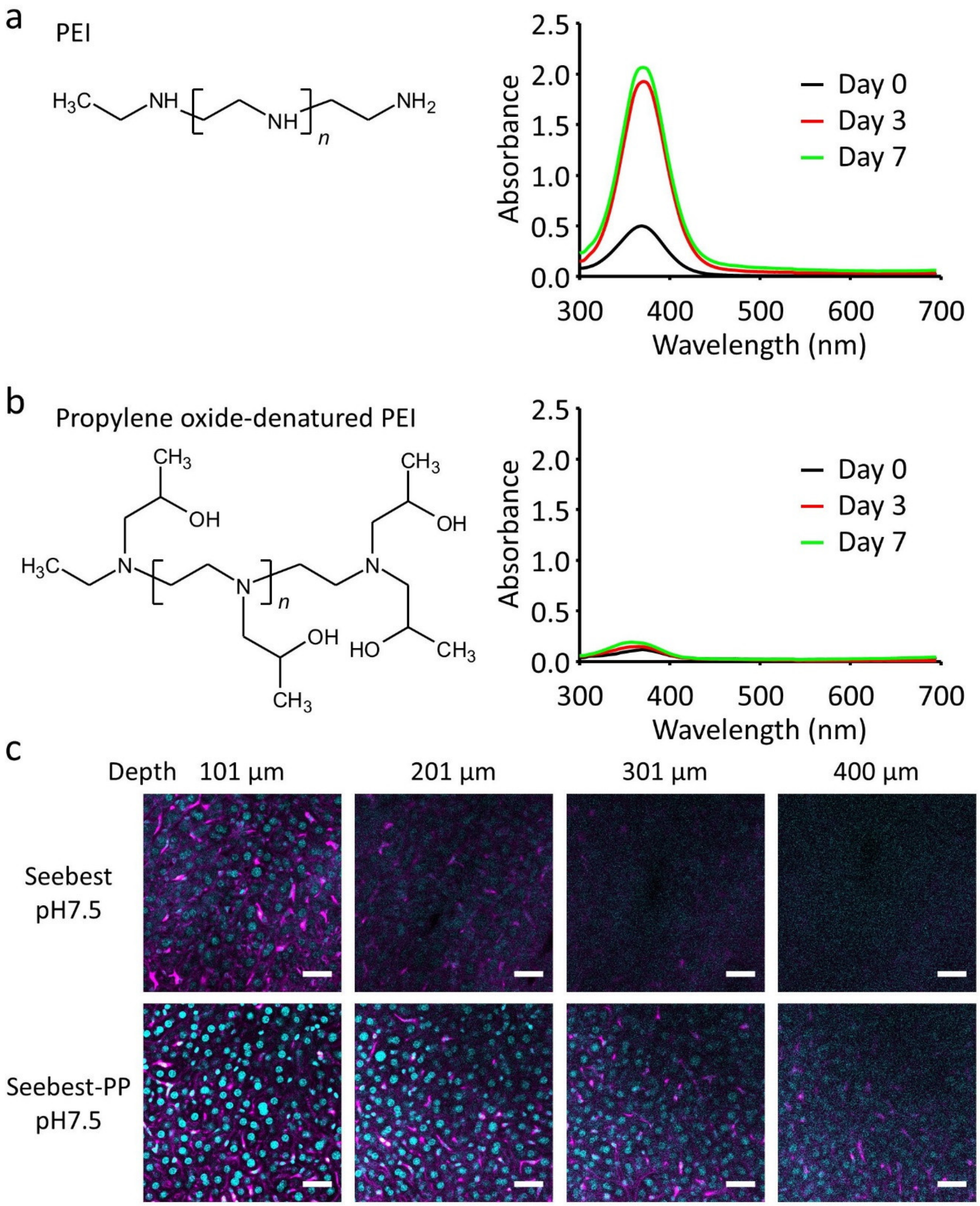

Figure S15. Improvement of the multicolor deep imaging potential of Seebest using propylene oxide-denatured PEI. (**a**) Chemical structure of PEI (branched form) and absorbance spectra of the Seebest solution (pH 7.5) at day 0, 3, and 7. (**b**) Chemical structure of propylene oxide-denatured PEI (branched form) and absorbance spectra of the Seebest-PP solution (pH 7.5) at day 0, 3, and 7. Seebest-PP solution comprising 8 M urea and 20 *w*/*v*% propylene oxide-denatured PEI was used. Seebest and Seebest-PP solutions were stored at 37°C. (c) Observable depths of nuclei stained with DAPI in the liver of DiI-perfused mice. DAPI (Cyan); DiI (Magenta). Fluorescence images were acquired as a z-stack at 1.247-µm intervals using a confocal microscope with a ×25 LD LCI Plan-Apochromat objective lens. Acquisition settings were as follows: zoom, 1.0; xy scaling, 0.664 µm; spectral emission filters (bandwidth), 409–521 and 550–670 nm; laser wavelengths, 405 and 543 nm for DAPI and DiI, respectively. The pH of Seebest and Seebest-PP was 7.5. Scale bars represent 50 µm. Each image was representative of three experiments.

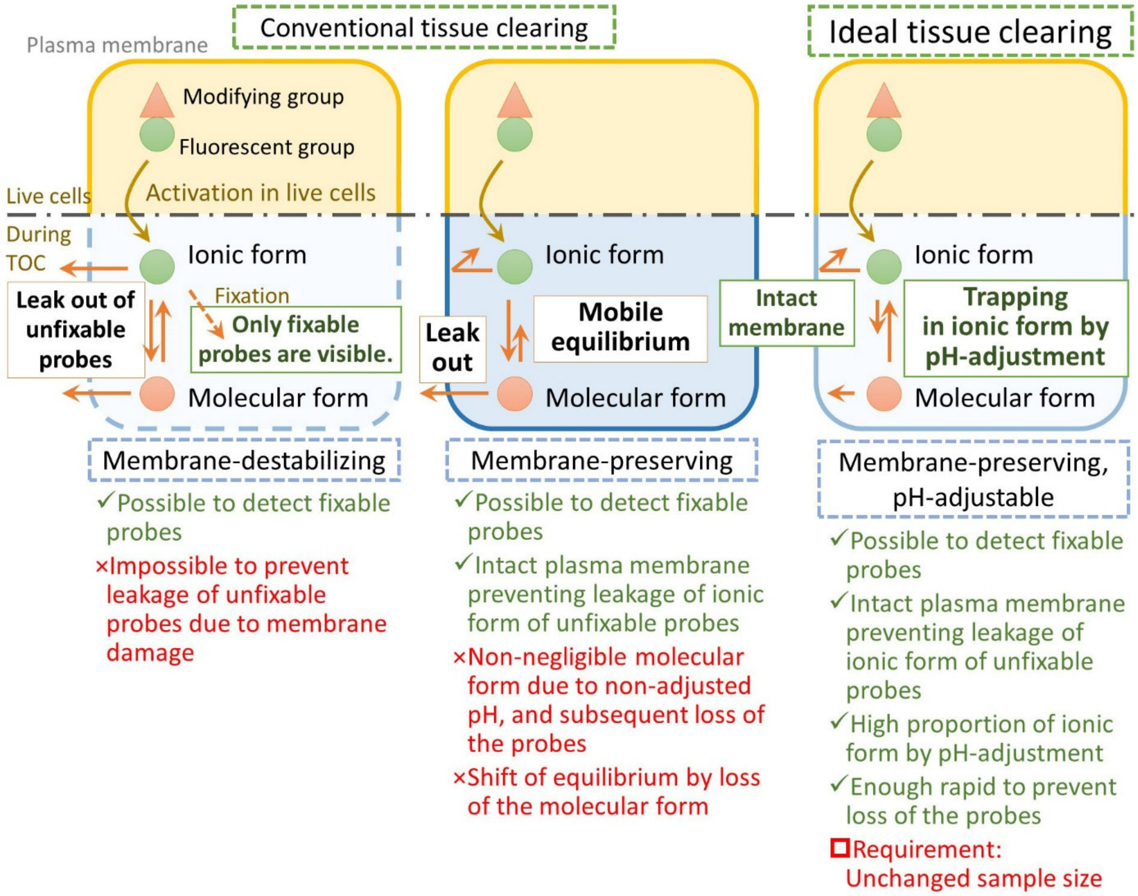

Figure S16. Schematic representation of minimizing the release of small fluorescent molecules from cells. Typically, fluorescent probes are modified by a penetration-enhancing group. In live cells, fluorescent probes are activated into their fluorescent form. This form is often an ionic form. Conventional membrane-destabilizing tissue clearing methods cannot retain the unfixable fluorescent probes because of a leaky plasma membrane. In conventional membrane-preserving tissue clearing methods, a non-negligible molecular form of the probes presents because of non-adjusted pH, subsequently losing the molecular form and leading to a shift in the equilibrium from ionic to molecular forms. This mobile equilibrium causes further loss of the probes. Ideal tissue clearing maximizes the proportion of the ionic form by pH adjustment and minimizes the loss of the probes, but requires unchanged sample size. Seebest slightly changed sample size during tissue clearing, and slightly lost the probes. But, Seebest can rapidly clear tissues, thus enabling observation before loss of the probes.

Table S1. Comparison of clearing speeds of various tissue clearing solutions using a tissue mimic on a plate.

Tissue clearing solutions	Slope of curves (Fig. S8) calculated using initial 30-min data			
	OD405	OD450	OD550	OD620
Seebest pH 11	−0.0455 ± 0.0037	−0.0372 ± 0.0025	−0.0372 ± 0.0030	−0.0365 ± 0.0026
CUBIC-1	−0.0344 ± 0.0056	−0.0295 ± 0.0043 #	−0.0300 ± 0.0039 #	−0.0290 ± 0.0038 #
CUBIC-2	−0.0342 ± 0.0065	−0.0330 ± 0.0035	−0.0323 ± 0.0030	−0.0308 ± 0.0026 #
Clear^{T2}	−0.0162 ± 0.0080 *, ###	−0.0184 ± 0.0049 *, ###	−0.0195 ± 0.0046 **, ###	−0.0196 ± 0.0044 **, ###
Sca*l*eSQ(0)	−0.0243 ± 0.0053 #	−0.0260 ± 0.0035 ##	−0.0267 ± 0.0032 ##	−0.0262 ± 0.0029 ##
FUnGI	−0.0537 ± 0.0008	−0.0483 ± 0.0003	−0.0460 ± 0.0005	−0.0440 ± 0.0007

Each value represents the mean ± S.E. of six experiments. Statistical comparisons were performed using Tukey's test. *$p < 0.05$ and **$p < 0.01$ vs. Seebest pH11 group. #$p < 0.05$, ##$p < 0.01$, and ###$p < 0.001$ vs. FUnGI group.

Video S1. Rotatory image for visualization of labile Fe^{2+} ions in the liver of ischemia/reperfusion-injured mice. The data were identical to Figure S8a. Deconvolution was performed using ImageJ software. Cyan, DAPI. Yellow, SiRhoNox-1.

Video S2. Rotatory image for relationship between transgene expression and ROS after hydrodynamics-based transfection to the liver. Cyan, transgene expression (ZsGreen1). Yellow, ROS (CellROX Deep Red).

Supplementary Methods

Absorbance of tissue homogenates. The vena cava of mice was cut under anesthesia, and then the brain, liver, and kidney were harvested. After addition of PBS (−) (4 μL/mg tissue), tissues were homogenized using a handheld tissue homogenizer (Omni TH, Omni Inc.). Obtained homogenates were mixed with tissue clearing solutions, and then absorbance was measured by a plate reader (Multiskan FC basic, Thermo Fisher Scientific Inc.).

Quantitation of tissue clearing speed. Tissue clearing speed was evaluated using a tissue mimic on a 96-well plate. A liver homogenate was mixed with bovine gelatin in PBS (−) at 50°C. The final concentrations of liver tissue and gelatin were 10 and 5 w/v%, respectively. After addition of 60 μL of the mixture to each well, the mixture was gellated in a refrigerator for 6 h. Then, 200 μL of 4% paraformaldehyde in PBS(−) was added to each well and the gel was fixed in a refrigerator for 24 h. Before adding a tissue clearing solution, each well was washed three times with PBS(−). After incubation with the tissue clearing solution at 37°C, absorbance was measured using a plate reader (Multiskan FC basic).

Monitoring of the tissue size change. To measure the volume of each tissue, a graduated cylinder (5 mL) was used. Briefly, the fixed liver specimen was cut to an appropriate size, and approximately 0.5 g of the fixed liver pieces were incubated with tissue clearing solutions. Three milliliters of tissue clearing solution was poured into the graduated cylinder, and then the liver sample was placed in the graduated cylinder. The change in the volume was measured immediately.

Visualization of brain parenchyma. To stain the brain parenchyma, a thick brain slice was prepared after DiI perfusion and fixation. The thick brain slice was immersed in DiD solution [0.16 mg/mL in PBS (−)]. The slice was immersed in Seebest (pH 11) for 1 day and then imaged using confocal microscopy.

Fluorescence spectroscopy. Fluorescence spectra and intensities of fluorescent dyes and proteins were measured by a spectrofluorometer (RF-6000, Shimadzu Corp.) after immersion in tissue clearing solutions.

Preparation of DiI liposomes. Egg lecithin, cholesterol, and DiI were dissolved in methanol at a weight ratio of 2:1:0.048. Then, a thin lipid film was obtained by a rotary evaporator and subsequently desiccated overnight. To obtain DiI liposomes, the thin lipid film was rehydrated in a 5% glucose solution at a final concentration of 0.4 mg/mL. To form small unilamellar vesicles, liposomes were extruded through polycarbonate membrane filters (100- and 50-nm pore sizes, 11 times for each pore size) using a commercially available instrument (Mini-Extruder, Avanti Polar Lipids, Inc.).

Cell culture. HepG2 cells were obtained from RIKEN (Tokyo, Japan). The cells were grown under standard conditions in Dulbecco's modified Eagle's medium (DMEM) supplemented with 10% foetal bovine serum (FBS) and penicillin G (100 U)/streptomycin (100 μg/ml) in a humidified atmosphere with 5% CO_2 at 37°C.

Labile Fe^{2+} ion probes. Labile Fe^{2+} ion probes SiRhoNox-1 and HMRhoNox-M were synthesized as reported previously [24,25]. Thirty minutes after intraperitoneal injection of SiRhoNox-1 (100 nmole) or HMRhoNox-M (100 nmole) together with CellROX Deep Red (125 μmole), liver ischemia/reperfusion injury was induced. After liver perfusion fixation, the liver was post-fixed overnight, washed twice with PBS (−), immersed in Seebest for 1 day, and then observed by confocal microscopy. For HepG2 cells, 2×10^4 cells/well were seeded into a 96-well glass-bottom plate and cultured for 24 h. The medium was replaced with FBS-free DMEM (100 μL), and 5 μL of 2 mM ferrous ammonium sulphate in water (final conc. 100 μM) were added to each well. Thirty minutes after addition, each well was washed with FBS-free DMEM, and then treated with 100 μL of 1 μM HMRhoNox-M in FBS-free DMEM for 30 min. After that, each well was washed with PBS (−), immersed in tissue clearing solution containing DAPI (5 μg/mL), and then observed by confocal microscopy.

Hydrodynamics-based *in vivo* transfection to the liver. Mice were injected with a plasmid DNA solution (10 μg/2.2 mL in saline, pZsGreen1-N1, Takara Bio Inc., Japan, 632448) within 5 sec via the tail vein. One hour prior to plasmid DNA injection, CellROX Orange (125 μmole) was injected intraperitoneally. Twelve hours after plasmid DNA injection, mice were intravenously injected with a DiD solution (4.8 mg/kg). Thirty minutes after DiD injection, mice underwent liver perfusion fixation. Liver specimen were cleared in Seebest (pH 8.5) and Sca*l*eSQ(0) for 1 day and then confocal microscopy was performed.

Immunohistochemistry after immersion with Seebest. An anti-α-tubulin antibody (B-7, for murine liver experiments) was obtained from Santa Cruz Biotechnology (Santa Cruz, CA, USA). An anti-α-tubulin antibody (DM1A, for HepG2 cell experiments) was obtained from Abcam (Cambridge, UK). Hilyte Fluor 647-labelled anti-α-tubulin antibody (200 μg/mL) was prepared using a HiLyte Fluor 647 Labeling Kit - NH_2 (Dojindo Molecular Technologies, Inc.). A liver specimen and HepG2 cells were immersed in Seebest (pH 7.5) for 1 and 3 days, respectively. The cleared liver was washed with PBS (−) for 1 day. The liver specimens were immersed in 10%, 20%, and 30% sucrose, and then frozen in Tissue-Tek O.C.T. compound (Sakura Finetek Japan). Frozen samples were sectioned at 40-μm thicknesses with a microtome (Retoratome REM-710; Yamato Kohki Industrial Co., Ltd., Saitama, Japan). Sections were permeabilized with 0.2% Triton X-100 in PBS (−), blocked with 1% bovine serum albumin in PBS (−), and then incubated with the Hilyte Fluor 647-labelled anti-α-tubulin antibody (B-7) at a dilution of 1:50. After 3 h of incubation, the section was washed five times with PBS (−), mounted with SlowFade Diamond reagent with DAPI (Thermo Fisher Scientific Inc.), and then observed by confocal microscopy. The HepG2 cells were washed with 2.5% BSA in PBS (−) for 7 hours, after that, staining with Hilyte Fluor 647-labelled anti-α-tubulin antibody (DM1A) was performed in similar fashion, and then observed by confocal microscopy.
